# A nanoparticle platform for combined mucosal healing and immunomodulation in inflammatory bowel disease treatment

**DOI:** 10.1016/j.bioactmat.2023.09.014

**Published:** 2023-10-11

**Authors:** Valentina Marotti, Yining Xu, Cécilia Bohns Michalowski, Wunan Zhang, Inês Domingues, Hafsa Ameraoui, Tom G. Moreels, Pieter Baatsen, Matthias Van Hul, Giulio G. Muccioli, Patrice D. Cani, Mireille Alhouayek, Alessio Malfanti, Ana Beloqui

**Affiliations:** a UCLouvain, Université catholique de Louvain, Louvain Drug Research Institute, Advanced Drug Delivery and Biomaterials, 1200 Brussels, Belgium; b UCLouvain, Université catholique de Louvain, Louvain Drug Research Institute, Bioanalysis and Pharmacology of Bioactive Lipids, 1200 Brussels, Belgium; c UCLouvain, Université catholique de Louvain, Institute of Experimental and Clinical Research, Laboratory of Hepato-Gastroenterology, 1200 Brussels, Belgium; d Cliniques universitaires Saint-Luc, Department of Hepato-Gastroenterology, Brussels, Belgium; e EM-platform, VIB Bio Imaging Core, KU Leuven, Campus Gasthuisberg, Herestraat 49, 3000 Leuven, Belgium; f UCLouvain, Université catholique de Louvain, Louvain Drug Research Institute, Metabolism and Nutrition Group, 1200 Brussels, Belgium; g UCLouvain, Institute of Experimental and Clinical Research, 1200 Brussels, Belgium; h WEL Research Institute, Avenue Pasteur, 6, 1300 Wavre, Belgium

**Keywords:** Nanocarrier, GLP-2, Teduglutide, KPV, Inflammatory bowel disease

## Abstract

Current treatments for inflammatory bowel disease (IBD) treatment consist of anti-inflammatory products. In this study, we sought to induce the physiological secretion of glucagon-like peptide 2, a peptide with intestinal growth-promoting activity, via nanoparticles while simultaneously providing with immunomodulation by tailoring the nanoparticle surface. To this end, we developed hybrid lipid hyaluronate-KPV conjugated nanoparticles loaded with teduglutide for combination therapy in IBD. The nanocarriers induced (or did not induce) immunosuppression depending on the presence (or absence) of a hyaluronan-KPV functionalization. This strategy holds promise as a nanoparticle platform for combined mucosal healing and immunomodulation in IBD treatment.

## Highlights

• We have demonstrated that lipid nanocapsules per se increased GLP-2 secretion *in vitro* and *in vivo* in a murine acute colitis model.
• We tested the mucosal healing and anti-inflammatory properties of the formulation, demonstrating no immunomodulatory effect of the nanocarriers.
• Hyaluronan-KPV functionalized teduglutide-loaded nanocarriers combined anti-inflammatory and mucosal healing properties upon intermittent administration.
• The pharmacological effect exerted by our nanocarriers induced sufficient stimulus as per providing with mucosal healing upon intermittent administration of our formulation.

## Graphical abstract

**Figure:**
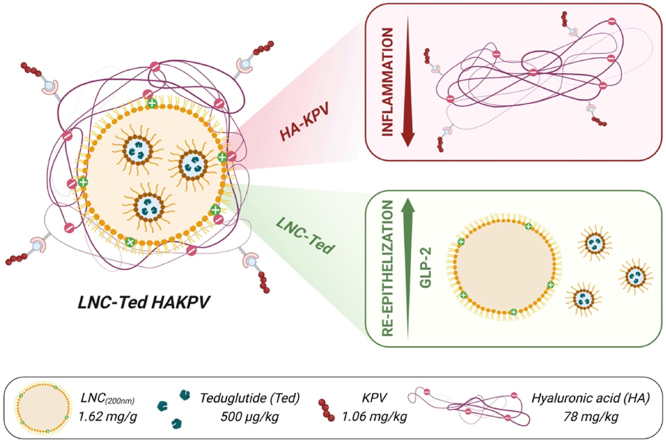

## 1 Introduction

Inflammatory bowel disease (IBD) is a systemic, autoimmune, relapsing-remitting chronic disease of the gastrointestinal system, with ulcerative colitis (UC) and Crohn's disease (CD) being the major forms of IBD [1]. Current marketed treatments for IBD, and most of the drugs in the pipeline, are anti-inflammatory products. There is an unmet need for treatments aimed at repairing the intestinal epithelial barrier to improve clinical outcomes [2].

Glucagon-like peptide 2 (GLP-2) is a 33 amino acid peptide cosecreted with GLP-1 from enteroendocrine L cells that has demonstrated intestinal growth-promoting activity [3]. However, GLP-2 is rapidly cleaved by the dipeptidyl peptidase IV (DPP-IV), constraining its intestinotrophic activity [4]. Teduglutide, a GLP-2 analog with prolonged half-life, is commercialized as a daily subcutaneous injection (Gattex®, Revestive®) for its use in short bowel syndrome (SBS) in patients who are dependent on parenteral support. A second long-acting GLP-2 analog, glepaglutide (Zealand Pharma), has just completed Phase III clinical trials for its use in SBS (clinical trials.gov identifier: NCT03690206) [5]. Owing to their trophic effects on the intestinal mucosa, GLP-2 analogs could be plausible mucosal healing candidates for IBD treatment [6], considering that the growth stimulation is specific to the gastrointestinal tract [7]. While the exogenous administration of GLP-2 has been proven to accelerate the intestinal growth of adenocarcinomas in murine models of colonic neoplasia [8], endogenous GLP-2 has not [9,10]. Thus, increasing native GLP-2 levels would be more convenient as a treatment over exogenous GLP-2 administration.

Our group has recently demonstrated that lipid nanoparticles can stimulate the secretion of GLP-1 from L cells [11]. Lipid nanocapsules (LNCs) charged with a GLP-1 analog could not only increase endogenous GLP-1 secretion but also simultaneously help to deliver the encapsulated synthetic peptide in the plasma, thereby increasing total plasma GLP-1 levels [12,13]. As GLP-2 is cosecreted with GLP-1 [3], we hypothesized that we could also stimulate the physiological secretion of GLP-2 and encapsulate a GLP-2 analog (teduglutide) within our nanocapsules to increase GLP-2 levels. Teduglutide exerts intestinal morphological effects in a dose-dependent manner [3,6]. Thus, increasing the concentrations of GLP-2/teduglutide could improve the efficacy of GLP-2 in the context of IBD while providing with a safer alternative to the exogenous administration of the peptide *per se*.

Although increasing GLP-2 levels might be efficient at restoring the gut barrier, its effect might not be sufficient to reduce IBD-associated inflammation [14]. Because different stages of IBD disease might need different treatment approaches, we aimed to develop a versatile nanoparticle platform that could provide with anti-inflammatory properties, or not, in combination with an intestinotrophic effect. However, most of the anti-inflammatory drugs used in IBD treatment (e.g., cortisone) are likely to cause systemic adverse effects. Thus, to endow our nanocarriers with these anti-inflammatory properties, we functionalized our nanocarriers with the Lys-Pro-Val (KPV) tripeptide via hyaluronic acid (HA) conjugation. Indeed, the oral administration of KPV has been shown to decrease proinflammatory cytokine expression in murine IBD models [15]. The complexation of these two molecules together (KPV and HA) has been previously shown to alleviate IBD-associated inflammation [16]. We hypothesized that we could retain the GLP-2 secretory effect of our nanocarriers while providing additional anti-inflammatory properties when needed.

To achieve therapeutic levels of the GLP-2 peptide in the chronic setting, prolonged or increased doses of the peptide would be needed, and this is a limitation that is hampering the use of the peptide in the context of IBD treatment in the clinics. The novelty of our study resides on the combination therapy of a re-epithelizing agent with an anti-inflammatory moiety within one sole nanoparticle. Our strategy provides with increased levels of GLP-2, in addition to anti-inflammatory effects. To achieve HA-KPV conjugation, we exploited a redox responsive (disulphide bond) linker using 4-(4,6-Dimethoxy-1,3,5-triazin-2-yl)-4-methylmorpholinium chloride (DMTMM·Cl), more easily scalable [17]. We chose a high MW HA (1 MDa) that has been described as anti-inflammatory [18]. Our goal was to develop a nanoparticle platform that would allow us to encapsulate a peptide and provide with a combination therapy efficient in chronic IBD treatment upon intermittent/short periods of administration.

To validate our hypothesis, in this study we evaluated lipid nanocapsule-mediated GLP-2 secretion and its effect in the context of IBD treatment in murine colitis via the oral route. We hypothesized that by orally administering lipid nanocapsules, we could increase endogenous GLP-2 secretion and plasmatic levels of teduglutide, while following the normal physiological pathway of the peptide. In the first step, we evaluated the ability of the formulation *per se* (unloaded) to induce GLP-2 secretion *in vitro* (L cells) and *in vivo* (murine colitis model). In a second step, we studied the mucosal healing effect of our nanocapsules loaded with teduglutide in the context of IBD treatment in two different acute IBD murine models, namely, the dextran sodium sulfate (DSS)- and trinitrobenzene sulfonic acid (TNBS)-induced IBD models [19]. To conclude, we evaluated the effect of hybrid lipid hyaluronate-KPV conjugated nanoparticles loaded with teduglutide for combination therapy in IBD in murine DSS-induced acute and chronic models of colitis.

## 2 Materials and methods

### 2.1 Materials

Labrafac® WL 1349 (caprylic/capric acid triglycerides) was kindly provided by Gattefossé (Saint-Priest, France). Lipoid® S75-3 (soybean lecithin at 69% of phosphatidylcholines and 10% of phosphatidylethanolamines) was purchased from Lipoid GmbH (Ludwigshafen, DE). Solutol® HS15 (mixture of free PEG 660 and PEG 660 12-hydroxystearate, Mw 870 Da), trinitrobenzene sulfonic acid (TNBS), sodium deoxycholic acid, trizma, *o*-dianisidine, hexadecyltrimethylammonium bromide (HTBA), 4-(4,6-Dimethoxy-1,3,5-triazin-2-yl)-4-methylmorpholinium chloride (DMTMM·Cl), sodium chloride (NaCl) were purchased from Sigma-Aldrich (St. Louis, USA). l,2-dioleoyl-3-trimethylammonium-propane (DOTAP) was purchased from Avanti Polar Lipids, Inc (Alabama, USA). Sodium hyaluronate (Mw 1 MDa) was purchased from Lifecore Biomedical LLC (Chaska, MN, USA), CysKPV was obtained from Synpeptide (Shanghai, China) and 2-(pyridyldithio) ethylamine was synthetized according to a previously described protocol [20]. Dulbecco's phosphate-buffered saline (DPBS) was provided by ThermoFisher Scientific (USA). Ethylenediaminetetraacetic acid (EDTA) was purchased from Thermo Scientific (BE). Protease inhibitor cocktail tablets (cOmplete mini, EDTA free) were purchased from Roche Diagnostics (Vilvoorde, BE). Triton X-100 was purchased from Carl Roth GmbH, DE. Sodium dodecyl sulfate (SDS) was purchased from Bio-Rad (BE). Potassium dihydrogen phosphate (KH_2_PO_4_) and hydrogen peroxide (H_2_O_2_) were obtained from Merck (Darmstadt, DE). Teduglutide was purchased from Creative peptides (NY, USA). A total GLP-2 ELISA kit, measuring both GLP-2 (1–33) and GLP-2 (3–33), was purchased from Eagle Biosciences, USA. A mouse citrulline ELISA kit was purchased from MyBiosource (MBS2602136). Dextran sulfate sodium (DSS) (Mw ∼40,000 Da) was purchased from TdB Consultancy (Uppsala, SE). Dipeptidyl peptidase IV (DPP-IV) inhibitor was purchased from Millipore (St. Charles, USA). All chemical reagents utilized in this study were of analytical grade.

### 2.2 Synthesis and characterization of HA-KPV

#### 2.2.1 Synthesis of HA-KPV

Sodium hyaluronate (100 mg; 0.248 mmol of (β,1 → 4)-d-glucuronic acid-(β,1 → 3)-N-acetyl-d-glucosamine repeat unit, 100 eq.) was dissolved in 20 mL of PBS buffer at pH = 7.4. After dissolution, DMTMM·Cl (34.33 mg, 0.124 mmol, 50 eq.) was added to the solution and stirred (250 rpm) for 90 min at room temperature (RT). Afterwards, 2-(pyridyldithio) ethylamine (PD) linker (16 mg; 0.072 mmol, 35 eq.) was added to the mixture. The reaction was stirred at RT for 48 h. The mixture was purified through precipitation in cold ethanol (99%, 4 °C, 3 × 50 mL). The final product precipitate was vacuum-dried overnight before freeze-drying. Lyophilized HA-PD (100 mg, 0.248 mmol, 100 eq.) was then resuspended in 20 mL PBS (5 mg/mL) and CysKPV (6.1 mg, 0.0137 mmol 5.5 eq.) was sequentially added and left to react overnight at RT under constant stirring. The mixture was purified by dialysis (Mw cutoff = 6–8 kDa) against Milli-Q water, and the final product was obtained after freeze-drying into a fluffy white powder and analyzed by ^1^H NMR and RP-HPLC.

HA-KPV: Yield: 81.6% (w/w); ^1^H NMR (D_2_O): *δ ppm* 4.6–4.3 (2H, bm), 3.9–2.9 (12H, m), 2.1–1.18 (3H, s), 0.9 (6 H, m); KPV loading (w/w): 2.5%.

#### 2.2.2 Chemical-physical characterization of HA-KPV

##### 2.2.2.1 NMR

HA-KPV conjugation was verified by ^1^H NMR spectra recorded with a Bruker Ultrashield Advance II 400 MHz instrument in D_2_O (5 mg/mL). The chemical shifts were reported as *δ* in parts per million (ppm) and were referenced against the residual solvent peak of D_2_O.

##### 2.2.2.2 HPLC

KPV loading was quantified by HPLC. The conjugated polymer was dissolved in Milli-Q water under extremely reducing conditions (10 mM DTT) and analyzed using a Shimadzu Prominence system (Shimadzu, Kyoto, Japan). A nucleosil C18 (Macherey-Nagel, Duren, Germany) (250 mm × 4.6 μm) column was used to separate the desired product. Then, 0.1% of trifluoroacetic acid (TFA) in water and acetonitrile (ACN) were used as mobile phases and applied in gradient mode (99% 0 min; 70% 15 min; 99% 20–30 min). The quantification method was set to a detection wavelength of 220 nm, a sample injection volume of 50 μL, and a flow rate of 1 mL/min. Free peptide content was assessed by means of a previously performed CysKPV calibration curve (concentration range of 10–200 μg/mL; R^2^ = 0.999) (limit of detection (LOD) 6.82 ± 1.4 μg/mL).

### 2.3 Preparation and characterization of lipid nanocapsules

LNCs were prepared by the phase inversion process first described by Heurtault et al. [21] To obtain 200 nm sized LNCs, Labrafac WL 1349, Peceol, Kolliphor HS15, NaCl, and Milli-Q water were mixed together at room temperature with either DOTAP or Lipoid to have positively or negatively surface charged LNCs, respectively. In addition, reverse micelles were prepared using Labrafac, Span 85 and teduglutide solution. Three temperature cycles of progressive heating/cooling were performed (from 68 to 50 °C) under constant stirring (220 rpm). Reverse micelles (RM) were incorporated during the last cycle when the temperature reached 64 °C under high-speed stirring (300 rpm). Cold Milli-Q water (4 °C) was added during the last cooling step at 60.5 °C. The obtained LNCs were then stored at 4 °C until use. The final composition for each LNC formulation is summarized in Supplementary Information Table 1S.

#### 2.3.1 Preparation of hyaluronate-KPV-coated lipid nanocapsules

To obtain 1 mL of Ted-loaded hyaluronate-KPV-coated lipid nanocapsules (LNC HAKPV) formulation, hyaluronate-KPV conjugated polymer was dissolved in 600 μL of Milli-Q water at increasing concentration values and added to 400 μL of positively charged LNC solution under vigorous stirring to obtain different DOTAP/HA ratios (from 1:0.5 to 1:20 (w/w)). The obtained hyaluronate-KPV-coated lipid nanocapsules (LNC HAKPV) were stored at 4 °C until use.

#### 2.3.2 Nanoparticle physicochemical characterization

##### 2.3.2.1 Particle size and ζ potential measurements

All the NPs were characterized in terms of size and polydispersity index (PdI) by dynamic light scattering (DLS) using a Zetasizer Nano ZS (Malvern Instruments Ltd., Worcestershire, U.K.). Zeta potential was measured using a Zetasizer Nano ZS (Malvern Instruments Ltd., Worcestershire, U.K.). Each measurement was performed in triplicate (10 μL of LNCs in 2 mL of Milli-Q water). The physicochemical properties of each LNC formulation are summarized in Supplementary Information Table 2S. Representative DLS graphs of the nanoparticles’ size and zeta potential are depicted in Supplementary Information Fig. 1S.

##### 2.3.2.2 Cryogenic transmission electron microscopy (cryo-TEM)

To further explore the morphology and size of LNC, LNC and LNC HAKPV samples were diluted 10× in Milli-Q filtered water. Then, 3.5 μL of a sample was deposited on a lacey grid, glow-discharged with a Leica ACE600 (Leica, Vienna, AT) coating unit, in a humidity controlled chamber of a Leica GP2 plunge-freezer. After back-blotting for 2 s, the grids were vitrified by plunging in liquid ethane close to its freezing point and stored under liquid nitrogen.

Samples were observed and imaged in a JEOL F200 (JEOL, Tokyo, JP) transmission electron microscope, equipped with a Gatan Continuum energy filter and K3 camera, using zero loss filtering with a slid width of 20 eV. Images were taken with pixel size of 0.53 nm and a maximum exposure dose of less than 60 electrons per Angstrom [2]. Representative cryo-TEM images of LNC and LNC HAKPV are depicted in Supplementary Information Fig. 1S.

##### 2.3.2.3 LNC encapsulation efficiency

The total amount of teduglutide was determined by breaking nanoparticles in methanol with a dilution factor of 1/20 (50 μL of LNC-Ted in 950 μL of the organic solvent) followed by strong vortexing. Free and encapsulated drugs were separated by ultrafiltration using Amicon® centrifuge filters (Mw cutoff: 100 kDa, 4000 × *g*, 4 °C, 20 min). Teduglutide in the filtrate (free drug) and the one dissolved in methanol (total drug) were quantified by high-performance liquid chromatography (HPLC, Shimadzu, Japan) using a Kinetex® EVO C18 column (100 Å, 2.6 μm, 150 × 4.6 mm) (Phenomenex, USA). Trifluoroacetic acid (0.05%) of in water and acetonitrile was used as the mobile phase and applied in gradient mode (90% 0 min; 10% 11 min; 90% 15 min). The UV wavelength was set at 220 nm, and the flow rate was 1 mL/min. Total and free drug content was assessed previously by performing a teduglutide calibration curve in both water and methanol (concentration range of 10–200 μg/mL; R^2^ = 0.999; teduglutide retention time 7.7 min; limit of detection (LOD) 1.19 ± 0.22 μg/mL).

The encapsulation efficiency (EE%) was then quantified using the following Eq. (1):

(1) $$EE\%=\frac{\left(total\;amount\;of\;Ted-free\;Ted\right)}{\left(total\;amount\;of\;Ted\right)}\cdot \;100$$

#### 2.3.3 *In vitro* stability studies in simulated gastric, intestinal, and colonic fluids

The *in vitro* stability of LNC HAKPV was tested in five different biomimetic media: gastric simulated fluid (GSF), fasted state-simulated gastric fluid (FaSSGF), fasted state-simulated intestinal fluid version 2 (FaSSIF–V2), fed state-simulated intestinal fluid (FeSSIF) version 2 (FeSSIF–V2) (biorelevant.com, UK; except for GSF). A detailed description of the composition of the simulated fluids used is presented in Supplementary Information in Table 3S. The influence of gastric, intestinal, and colonic conditions on nanocapsule stability was evaluated based on the nanocapsule size and the PDI. LNC HAKPV was incubated in the different media at 37 °C (10 μL of nanocapsules in 1 mL of media) under gentle stirring. At predetermined time intervals (0, 0.5, 1 and 2 h for stimulated gastric media and 0, 0.5, 1, 3 and 6 h for stimulated intestinal and colonic media), samples were withdrawn and then analyzed by DLS. The *in vitro* stability results are depicted in Supplementary Information in Fig. 2S.

### 2.4 *In vitro* GLP-2 secretory studies in murine L cells (GLUTag cells)

The intestinal murine L cell line GLUTag was kindly provided by Dr. Daniel J. Drucker (University of Toronto, Canada). GLUTag cells (murine L cells) were used from passages 17 to 28. Cells were grown in DMEM GlutaMAX supplemented with 10% (v/v) inactivated FBS and 1% (v/v) PEST (complete DMEM), at 37 °C in a 5% CO_2_/95% air atmosphere. For secretory studies, GLUTag cells (1.8 × 10^5^ cells/well) were seeded into Matrigel™-coated (10 μL/mL of medium) 24-well cell culture plates and allowed to adhere for 24 h. The next day, the cells were treated for 2 h with LNCs. The concentrations of the nanoparticles were based on the cytotoxicity study results (as previously described in refs. [11,22]). All experiments were conducted in complete DMEM medium in the presence of DPP-IV inhibitor at a final concentration of 50 μM (Millipore, St. Charles, MO, USA). Total GLP-2 concentrations were determined with an ELISA kit (Eagle Biosciences, Inc., USA).

### 2.5 *In vivo* studies

All experimental protocols were approved by and performed in accordance with the local animal committee (2018/UCL/MD/045 and 2021/UCL/MD/055) and as specified by the Belgian Law of 29 May 2013 on the protection of laboratory animals.

#### 2.5.1 Proof-of-concept of lipid nanocapsule-mediated GLP-2 secretion *in vivo* in a murine DSS-induced colitis model

Six-week-old male C57BL/6J mice (20–25 g) were purchased from Janvier Laboratories, France. Animals were housed under standard conditions and supplied with food and tap water *ad libitum* for 2 weeks. The animals were randomly divided into three groups (8 mice per group): vehicle-treated control group, vehicle-treated DSS control group and LNC-treated DSS group (unloaded nanocapsules). Colitis was induced via the administration of drinking water containing 3% (w/v) DSS for 5 consecutive days. Mice were orally administered 100−150 μL of nanocapsule suspension for three consecutive days (on days 5, 6 and 7), corresponding to a 1.62 mg/g nanocapsule dose. Untreated mice received drinking water instead. Mice were fasted for 6 h prior to oral gavage. On Day 7, blood samples were withdrawn from the tip of the tail vein using heparinized capillary tubes (60 μL) at 60 and 180 min after oral administration and from the portal vein at the time of sacrifice. Samples were collected in the presence of DPP-IV inhibitor (20 μL per mL of blood) and maintained on ice. Immediately after the studies, blood samples were centrifuged (1500 × *g*, 10 min at 4 °C), and the plasma was kept frozen at −80 °C until analysis. The total GLP-2 plasma levels were quantified by ELISA following the manufacturer's instructions (YK142, Eagle Biosciences, USA). Plasma citrulline concentrations (mucosal healing marker [23]) were measured in the portal vein using an ELISA kit (MyBiosource) 3 h post-LNC administration.

#### 2.5.2 Pharmacokinetics study

Six-week-old male C57BL/6J mice (20–25 g) were purchased from Janvier Laboratories, France. Animals were randomly divided into three groups (8 mice per time point) and housed in a controlled environment (room temperature of 23 ± 2 °C, 12 h daylight cycle) with *ab libitum* food intake and sterile water. After two weeks of acclimation, prior to the experiments, mice were fasted overnight with free access to sterile Milli-Q water. Teduglutide in solution, LNCs and LNC-Ted were orally administered at a 500 μg/kg Ted dose (8 mice per group), and corresponding LNC concentrations. Teduglutide was also subcutaneously administered at a 50 μg/kg dose. At different time points (0, 0.5, 1, 1.5, 2, 4 and 6 h), blood samples were collected from the tip of the tail vein in the presence of a DPP-IV inhibitor. Blood samples were then centrifuged (1500 × *g*, 10 min at 4 °C), and the teduglutide plasma concentration was quantified using an ELISA kit (YK142, Eagle Biosciences, USA). The pharmacokinetic parameters were analyzed using PKSolver [24].

#### 2.5.3 In vivo evaluation of teduglutide-loaded lipid nanocapsule-mediated GLP-2 secretion therapeutic effect in acute murine colitis models

Six-week-old male C57BL/6J mice (20–25 g) were purchased from Janvier Laboratories, France. Animals were housed under standard conditions and supplied with food and tap water *ad libitum* for 2 weeks. After this acclimation period, the mice were subjected to DSS or TNBS treatments to induce colitis. In the DSS-induced colitis model, colitis was induced by administration of drinking water containing 3% (w/v) DSS for 5 consecutive days, and colonic inflammation was assessed 7 days after the beginning of DSS treatment. In the TNBS-induced colitis model mice were anesthetized with isoflurane. TNBS (100 mg/kg, in 0.9% NaCl-ethanol, 50:50, v/v) was intrarectally administered (50 μL) into the colon using a cannula inserted 4 cm from the anus. Colonic inflammation was assessed 4 days after the beginning of TNBS treatment. The mice were administered both treatments daily (7 consecutive days in the DSS group and 3 consecutive days in the TNBS group). The animals were divided into the following five groups (8 mice per group): vehicle-treated control group (healthy mice), vehicle-treated DSS group, DSS + Ted group, DSS + LNC group and DSS + LNC-Ted (500 μg of Ted/kg; 1.62 mg/g LNC dose). All animals were treated every day at the same time (starting at 16:00 p.m.) without fasting. The untreated control group and the DSS control group received drinking water instead of the formulation. At Day 7 (DSS) or Day 4 (TNBS), the animals were treated on the same day. Mice were anesthetized with isoflurane, and blood was collected from the portal and tail veins 1 h postadministration. Then, the mice were sacrificed, and colon samples were collected for evaluation of the severity of colitis.

#### 2.5.4 In vivo evaluation of LNCs conjugated with HA or HAKPV (loaded or unloaded with Ted) effect in an acute DSS-induced murine colitis model

Six-week-old male C57BL/6J mice (20–25 g) were purchased from Janvier Laboratories, France. Animals were housed under standard conditions and supplied with food and tap water *ad libitum* for 2 weeks. After this acclimation period, the mice were subjected to DSS treatment for the induction of colitis, as in the previous *Study 3*. The animals were divided into the following seven groups (8 mice per group): vehicle-treated control group (healthy mice), vehicle-treated DSS group, DSS + Ted group, DSS + LNC HA group, DSS + LNC-Ted HA, DSS + LNC HAKPV and DSS + LNC HAKPV (500 μg of Ted/kg; 1.62 mg/g LNC dose; 1.06 mg of CysKPV/kg; 77.96 mg of HA/kg). All animals were treated for 6 consecutive days at the same time (starting at 16:00 p.m.) without fasting. The control group and the DSS control group received drinking water instead of the formulation. On Day 7, the mice were anesthetized with isoflurane, and blood was collected from the portal and cava veins. The mice were not treated on Day 7. After blood withdrawal from the portal and cava veins, the mice were euthanized, and colon samples were collected to evaluate the severity of colitis.

#### 2.5.5 In vivo evaluation of hybrid lipid hyaluronate-KPV conjugated nanoparticles in a DSS-induced chronic colitis model

Six-week-old male C57BL/6J mice (20–25 g) were purchased from Janvier Laboratories, France. Animals were housed under standard conditions and supplied with food and tap water *ad libitum* for 2 weeks. After this acclimation period, the mice were subjected to 3 cycles of 3% (v/v) DSS treatment to induce colitis. Each cycle lasted for 7 days with 14-day wash-out periods between cycles [19]. The animals were divided into the following six groups (8 mice per group): vehicle-treated control group (healthy mice), vehicle-treated DSS group, DSS + Ted group, DSS + LNC group, DSS + LNC-Ted and DSS + LNC HAKPV (500 μg of Ted/kg; 1.62 mg/g LNC dose; 1.06 mg of CysKPV/kg; 78.88 mg of HA/kg). All animals were administered DSS for 3 consecutive days at the same time (starting at 16:00 p.m.) without fasting on Days 5, 6 and 7 of the DSS cycle. The untreated control group and the DSS control group received drinking water instead of the formulation. At Day 50, the mice were anesthetized with isoflurane, a colonoscopy was conducted to assess the severity of the disease, and then blood was collected from the portal and cava veins. The mice were not treated on the day of euthanasia. After blood withdrawal, the mice were euthanized, and colon samples were collected to evaluate the severity of colitis.

#### 2.5.6 Myeloperoxidase activity (MPO)

The colonic tissue was placed in a 1.5 mL Eppendorf tube with 500 μL of HTAB buffer (0.5% HTAB in 50 mM potassium phosphate buffer (KH_2_PO_4_ 50 mM), pH 6) on ice and gently homogenized (Branson 450 digital sonifier®, 30% amplitude, 10 s) while kept on ice. The homogenates were centrifuged at 20,000 × *g* for 10 min at 4 °C. The supernatant (7, 14 or 21 μL) was added to 96-well plates (Nunc, Roskilde, DK) together with 200 μL of the MPO analysis solution (50 mM potassium phosphate buffer pH 6 containing 0.167 mg/mL *o*-dianisidine and 500 ppm hydrogen peroxide). The MPO activity in the supernatant was measured with a spectrophotometer (SpectraMax® iD5, Molecular Devices, LLC, USA) at 460 nm for 30 min. The results were expressed as MPO units per gram of tissue, and one unit of MPO activity was defined as the amount that degrades 1 mmol/min of hydrogen peroxide at 25 °C [25,26]. The concentrations were normalized to the protein content, quantified with a Pierce™ BCA Protein Assay Kit (Sigma-Aldrich, St. Louis, USA).

#### 2.5.7 Cytokine quantification by ELISA

Colon homogenates were directly prepared from frozen tissue samples in ice-cold lysis buffer (water with 500 nM NaCl, 2 mM EDTA, 1% Triton X-100, 0.5% sodium deoxycholic acid, 0.1% SDS, 50 mM Tris HCl and 1 tablet of protease inhibitor/mL of solution) by sonication (Branson 450 digital sonifier®, 30% amplitude, 10 s) (15 mg tissue/100 μL of lysis buffer). Samples were subsequently centrifuged at 20,000 × *g* for 10 min. The supernatant was retrieved and kept at −80 °C until further analysis, and the pellet was discarded. The concentrations of the following colonic proinflammatory cytokines and chemokines were determined with a V-PLEX Plus Mouse Cytokine 19-Plex ELISA kit (MesoScale) following the manufacturer's instructions using a MESO QuickPlex SQ 120 plate reader (MesoScale): *IFN-γ, IL-1β, IL-2, IL-4, IL-5, IL-6, IL-9, KC/GRO, IL-10, IL-12p70, Il-15, IL-*17AF *IL-27p28/IL-30, IL-33, IP-10, MCP-1, MIP-1α, MIP-2* and *TNF-α*. The concentrations were normalized to the protein content, quantified with a Pierce™ BCA Protein Assay Kit (Sigma-Aldrich, St. Louis, USA).

#### 2.5.8 RNA preparation and real-time qPCR analysis

Total RNA from colon tissues was extracted using TriPure reagent (Roche) following the manufacturer's instructions as described previously [27]. RNA was then purified using the Lithium Chloride as described by Viennois et al. [28] cDNA was synthesized using the Promega GoScript Reverse Transcription kit (Promega Benelux BV). Briefly, the reaction involved three steps: primer annealing at 25 °C followed by DNA polymerization at 42 °C for 1 h and finally enzyme deactivation at 70 °C for 15 min. qPCR was conducted using the Promega GoTaq qPCR Master Mix, with a QuantStudio™ 3 Real-Time PCR System (Thermo Fisher Scientific). Specific primer sequences were designed to exclusively amplify the genes of interest. qPCR included 45 amplification cycles consisting of the following steps: denaturation of the template cDNA at 95 °C for 3 s, annealing of the primers to the target template at 60 °C for 26 s and extension at 72 °C for 10 s. For each sample, PCR was run in duplicate during the same run. Postamplification melting curve analysis was used to ensure reaction specificity. Data are normalized to the 60S ribosomal protein L19 (RPL19) used as a reference gene. RPL19 mRNA expression was not affected by any of the conditions. The results were expressed as a percentage of the control group using the “delta-delta Ct” method [29]. The primer sequences used are listed in the table below.

**Table:** 

	Primers (5′–3′)	
Gene	Forward	Reverse
Rpl19	GAAGGTCAAAGGGAATGTGTTCA	CCTTGTCTGCCTTCAGCTTGT
Foxp3	GTTCCTTCCCAGAGTTCTTCC	CATCGGATAAGGGTGGCATAG
Muc2	TGACGAGTGGTTGGTGAATG	GAATAGGCTGTCCATGATGAGG
Tjp1 (ZO1)	TCATAGATCAGGATAAACATGC	GCTTAGAGTCAGGGTTAAGG
Ocln (Occludin)	AGGACTGGGTCAGGGAATATC	CGTCTAGTTCTGCCTGTAAGC
Lgr5	ACCCGCCAGTCTCCTACATC	GCATCTAGGCGCAGGGATTG

#### 2.5.9 Histological scoring

For histological scoring, small segments of the colon were fixed in 4% buffered formalin overnight, washed with 70% ethanol and subsequently embedded in paraffin. Two sets of 3 serial sections were cut 200 μm apart, and 6 sections were evaluated for each mouse. Sections were stained with hematoxylin–eosin, and histological scores were determined in a double-blind manner by two different researchers. The scoring procedure for the assessment of the severity of the disease was conducted following the histopathologic indices described by Koelink et al. [30] The histological scoring items included the presence of inflammatory infiltrate, goblet cell loss, crypt density, crypt hyperplasia, muscle thickening, submucosal inflammation, crypt abscess and ulceration, with each parameter being graded between 0 and 3.

#### 2.5.10 High-resolution colonoscopy in live mice

*In vivo* mouse colonoscopy was performed with a Coloview® colonoscopy system (Karl Storz, Tuttlingen, DE) in the chronic DSS study. On the final day of the study, mice were anesthetized (isoflurane) and examined with a mini endoscope. The endoscope was entered through the anus, and the colon was insufflated with an air pump. Several videos were recorded for each mouse, and images were captured from the videos. The colitis score (murine endoscopic index of colitis severity, MEICS) was scored as described by Becker et al. [31] focusing on five parameters (thickening of the colon, changes in the vascular pattern, visible fibrin, granularity of the mucosal surface and stool consistency), with each parameter being graded between 0 and 3 depending on the severity of the disease. The colonoscopy grading was performed with the help of a gastroenterologist blinded for the experimental treatment (T. Moreels, Saint-Luc Hospital, Brussels) in a single-blind study.

### 2.6 Statistical analysis

The statistical analysis was conducted using the GraphPad Prism 9 program (CA, USA). The Grubbs test for outlier detection was performed for each group to support any exclusion. Statistical analyses were conducted using two-way or one-way ANOVA followed by Tukey's post hoc test for comparisons among multiple groups according to the result of the Brown-Forsythe test of homogeneity of variances. If the variance differed significantly between groups, a nonparametric test was performed. The *t*-test or Mann-Whitney test was conducted to analyze differences between two groups. The results are expressed as the mean ± standard error of the mean (SEM). A difference of *P < 0.05 was considered statistically significant.

## 3 Results and discussion

### 3.1 Lipid nanocapsules induce GLP-2 secretion *in vitro* and *in vivo*

**Fig. 1:**
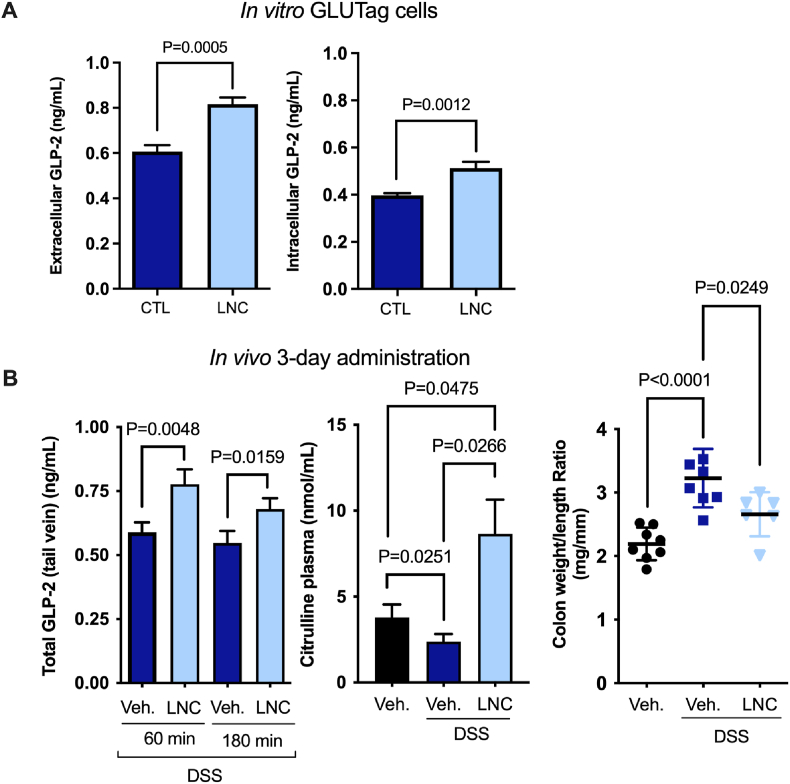
Lipid nanocapsules induce GLP-2 secretion *in vitro* and *in vivo*. A) Extracellular and intracellular GLP-2 concentrations in GLUTag cells after 2 h of coincubation with LNCs (n = 9–12). B) On the left, total GLP-2 plasma levels in DSS-treated mice 60 and 180 min after the oral administration of LNC on Day 7, following a daily 3-day administration of LNC from Day 5 (n = 8). In the middle, the plasma citrulline concentrations measured 3 h post-LNC administration (control healthy mice, DSS-treated mice and LNC-treated mice under DSS treatment) (n = 5–7). On the right, the colon weight/length ratios measured after a 3-day administration to DSS-induced colitis mice (n = 7–8). *P* values in (A, B) were determined by one-way ANOVA, Student's *t*-test or Mann-Whitney test (mean ± SEM). CTL: control; Veh: vehicle.

We hypothesized LNCs could induce endogenous GLP-2 secretion without any drug incorporated based on our previous studies. *In vitro*, LNCs significantly increased GLP-2 secretion and intracellular concentrations in murine L cells (Fig. 1A). *In vivo*, mice were subjected to 3% (w/v) DSS in the drinking water for five consecutive days and were orally administered drinking water or LNC on Days 5, 6 and 7. The aim was to evaluate the effect of LNCs on GLP-2 secretion once the inflammatory symptoms appeared in the murine DSS model. Hence, we administered the nanocapsules from Day 5, considering that after this period, the disease had already been established. The GLP-2 plasma concentrations of untreated and LNC-treated mice at 60 and 180 min after oral administration on Day 7 are depicted in Fig. 1B. GLP-2 plasma levels were significantly higher in LNC-treated mice than in DSS-treated mice (*P < 0.05). Notably, GLP-2 levels are already increased in IBD [6]. In addition to GLP-2, we measured plasmatic citrulline levels as a mucosal healing marker. This proof-of-concept study demonstrated our hypothesis that LNCs could induce endogenous GLP-2 secretion *in vitro* and *in vivo* in the context of IBD. Additionally, we observed increased citrulline levels in mice treated with LNCs compared to untreated mice (Fig. 1B). Inflamed colons are shorter and heavier, and thus, this ratio is increased in colitis. We further confirmed a decrease in the colon weight/length ratio in mice treated with our nanocapsules (Fig. 1B). Altogether, these data encouraged the use of these nanocapsules to restore the intestinal mucosa in IBD treatment. To our knowledge, this is the first time that lipid-based nanocarriers have been exploited to induce GLP-2 secretion *in vivo*.

### 3.2 Teduglutide-loaded LNCs induce mucosal healing without immunomodulation in acute murine colitis

To evaluate whether LNC-mediated GLP-2 levels could be therapeutically relevant, we evaluated the trophic effect of our nanocapsules in two different acute colitis models. There is no single animal model that recapitulates all of the pathogenic and clinical features of human IBD. Thus, we chose DSS and TNBS acute colitis models because these are widely used models, especially for evaluating novel therapeutic agents. Both models combined are believed to provide complementary information on the efficacy of the formulation. The DSS model is particularly useful for studying the innate immune response, and TNBS is particularly useful for studying the T helper cell-dependent mucosal immune response [32]. In both acute models, we opted for daily administration to evaluate the effect of the formulation after repeated doses. We also administered the formulations on the day of the sacrifice to evaluate the ability of the formulations to induce GLP-2 secretion following this regimen of administration in the two models.

In current IBD treatments based on anti-inflammatory drugs, mucosal healing is secondary to a decrease in inflammation. We hypothesized that we could induce the same effect the other way around. Our premise was that we could increase GLP-2 signaling by combining the GLP-2 levels exerted by the nanocarriers with teduglutide plasmatic levels. By increasing GLP-2 levels, we would be able to induce re-epithelization and consequently reduce inflammation.

**Fig. 2:**
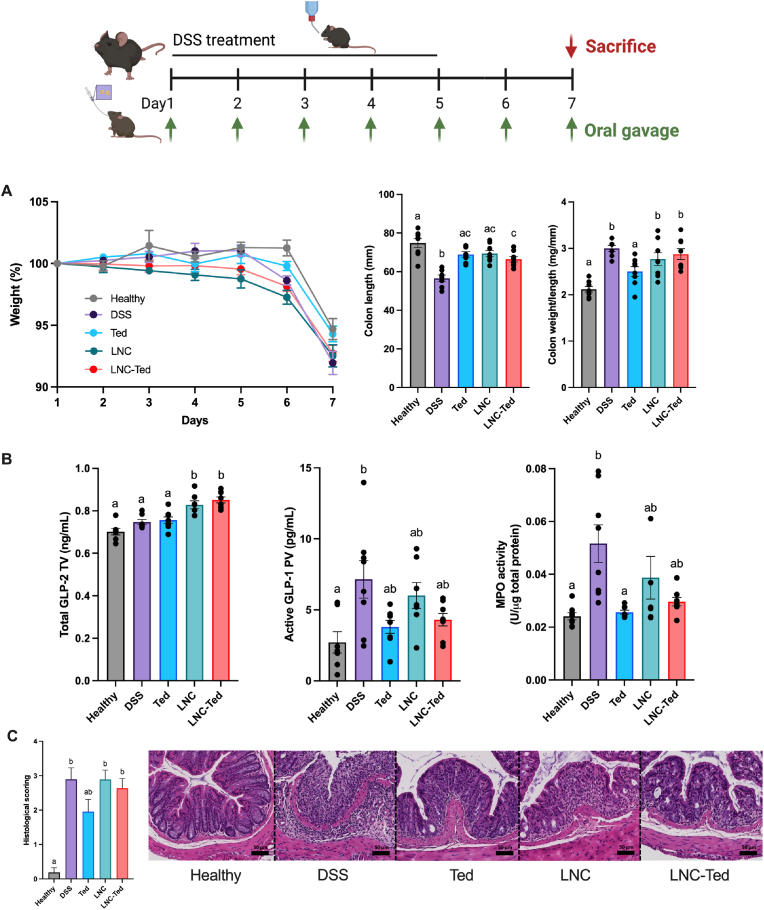
Lipid nanocapsule-mediated GLP-2 secretion effect in a DSS-induced acute colitis model. A) Weight loss (%), colon weight/length ratio and colon length (mm) (n = 8). B) Total GLP-2 plasma levels (ng/mL) (tail vein), active GLP-1 levels (pg/mL) and MPO activity (U/μg total protein) (n = 8). GLP-2 and GLP-1 levels were measured 1 h postoral administration of LNCs on the day of sacrifice (Day 7) (TV: tail vein; PV: portal vein). C) Histological scoring assessed following a double-blinded analysis per sample (n = 16) and representative colon H&E staining images (scale bar: 50 μm). Data with different superscript letters are significantly different (*P < 0.05) according to one-way analysis of variance followed by Tukey's post hoc test (A, B (except MPO)) or Kruskal-Wallis test followed by Dunn's post hoc test (B (MPO), C) (mean ± SEM).

In the DSS model, we followed a regimen consisting of a 7-day daily administration of nanocapsules loaded with teduglutide or nanocapsules and teduglutide alone and compared their effects on both healthy and DSS-treated control groups. We observed no differences in body weight between the groups on Day 7 (Fig. 2A). Despite observing increased GLP-2 plasmatic levels in LNC-treated mice (Fig. 2B), no changes in the colon weight/length ratio were observed. Considering nanoparticle-treated groups, the colon length was not significantly different compared to the healthy mice only in the case of unloaded-LNCs (Fig. 2A). We also evaluated active GLP-1 levels in the portal vein to evaluate whether there was a correlation between active GLP-1 secretion and GLP-2 secretion (Fig. 2B). In this case, active GLP-1 levels were increased in the DSS-treated mice but not in the rest of the groups. Notably, active GLP-1 was measured in the portal vein and total GLP-2 in the cava vein; thus, the kinetics would be expected to be different. Overall, we did not observe significant differences between LNC- or LNC-Ted-treated nanocarriers in the acute DSS model.

**Fig. 3:**
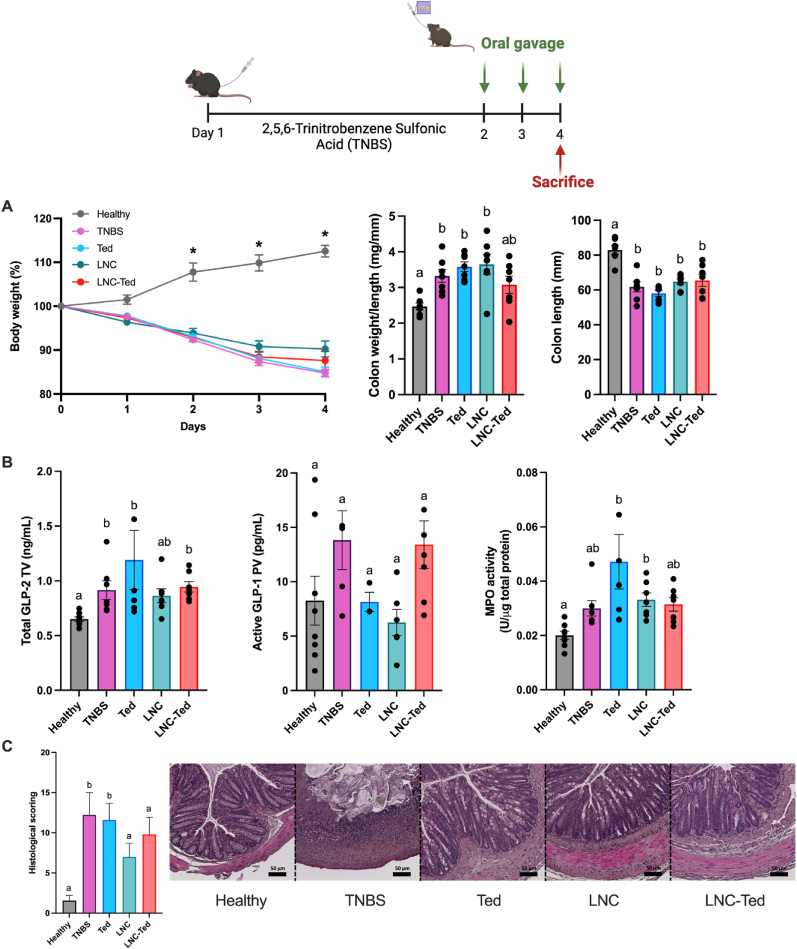
Lipid nanocapsule-mediated GLP-2 secretion effect in a TNBS-induced acute colitis model. A) Weight loss (%), colon weight/length ratio and colon length (mm) (n = 7–8) (*P < 0.05 Healthy *vs*. TNBS-treated groups. B) Total GLP-2 plasma levels (ng/mL) (tail vein) (n = 6–8), active GLP-1 levels (pg/mL) (n = 5–8, except for Ted n = 3) and MPO activity (U/μg total protein) (n = 7–8). GLP-2 and GLP-1 levels were measured 1 h postoral administration of LNCs on the day of sacrifice (Day 4) (TV: tail vein; PV: portal vein). C) Histological scoring assessment following a double-blinded analysis per sample (n = 14–16) and representative colon H&E staining images (scale bar: 50 μm). Data with different superscript letters are significantly different (*P < 0.05) according to one-way analysis of variance followed by Tukey's post hoc test (A) or Kruskal-Wallis test followed by Dunn's post hoc test (B, C) (mean ± SEM).

We anticipated that the gastric instability of teduglutide would block any *in vivo* effect of the peptide administered as a solution. However, we observed a colon weight/length ratio comparable to that of the healthy untreated group (Fig. 2A), decreased myeloperoxidase (MPO) activity (mirroring neutrophil infiltration) (Fig. 2B) and improved histological scoring (Fig. 2C) compared to the vehicle-treated DSS group. Indeed, the teduglutide-treated group presented histological results similar to that of healthy mice when compared to the rest of the treated mice (Fig. 2C), although all treated mice presented a disrupted and/or inflamed epithelial structure. This effect of teduglutide alone was not observed in the TNBS-induced colitis model (Fig. 3), which exhibited the opposite effect.

With regard to the TNBS model, we followed a 3-day administration regimen starting the day after the rectal administration of the TNBS solution. All TNBS-treated groups exhibited a significantly decreased body weight compared with the healthy control group over the treatment period, with no significant difference between the treated groups (Fig. 3A). In contrast to the effect observed in the DSS model, LNC-Ted-treated mice exhibited a decreased colon weight/length ratio without inducing an increase in colon length (Fig. 3A). However, the ratios were not significantly different from those of the control groups (healthy and TNBS). The MPO activity levels in the LNC-Ted-treated group were comparable to those in the healthy control group but not significantly different from those in the rest of the treated groups or the TNBS-treated group (Fig. 3C). Regarding GLP-2, while TNBS-induced colitis resulted in increased GLP-2 levels, the administration of the nanocarriers (LNC, LNC-Ted) had no significant effect on these levels (Fig. 3B). No differences were observed regarding active GLP-1 levels between groups, but there was a large variability within the control groups that might have impaired the determination of significant differences between the groups (Fig. 3B). Both LNC-treated groups, loaded and unloaded, exhibited a significantly reduced histological score compared to the TNBS- or teduglutide-treated groups (Fig. 3C).

We did not observe any difference in pro-inflammatory cytokine or chemokine levels between the LNC-treated groups (loaded or unloaded) when compared to the vehicle-treated DSS (Supplementary Information, Fig. 3S) or vehicle-treated TNBS (Supplementary Information, Fig. 4S) groups. Conversely, Ted-treated mice exhibited decreased TNF-α and IL-1β levels compared to vehicle-treated DSS mice (Supplementary Information, Fig. 3S) but not in TNBS-treated mice (Supplementary Information, Fig. 4S). We also evaluated the mRNA expression of gut immunity (*FoxP*3, *CD3g*), cell renewal (*Lgr5*) (B) and gut permeability (*ZO1*, *Ocln*, *Muc2*) biomarkers in the colon following acute treatment in both the DSS-induced colitis model (Supplementary Information, Fig. 5S) and the TNBS-induced colitis model (Supplementary Information, Fig. 6S). We observed no differences in the mRNA expression of these biomarkers between LNC-Ted-treated mice and the control groups subjected to DSS or TNBS (*P* > 0.05), except for *FoxP3* mRNA expression in the DSS-treated mice (*P < 0.05) (Supplementary Information, Fig. 5S). Ted-treated DSS mice exhibited decreased levels of *CD3g* compared to vehicle-treated DSS mice (Supplementary Information, Fig. 5S), which was not the case when comparing Ted-treated versus vehicle-treated mice in the TNBS model (Supplementary Information, Fig. 6S). Altogether, we observed no immunomodulatory effect exerted by lipid nanocapsules in either of the two acute colitis models (DSS and TNBS).

We also evaluated the plasma levels of teduglutide and GLP-2 upon oral administration to healthy mice (Supplementary Information, Fig. 7S). We used an ELISA kit for total GLP-2 quantification, which presented a high specificity to mouse GLP-2 and exhibited no cross reactivity with mouse glucagon and mouse GLP-1 [33]. We were not able to distinguish between endogenous GLP-2 exerted by the nanoparticles alone or the plasmatic levels absorbed teduglutide, probably due to their similar molecular formula (Supplementary Information, Fig. 7S). LNC and LNC-Ted showed an almost superposing GLP-2 profile.

Considering that we might need longer periods of administration to observe a complete re-epithelization, and taking into account that we did not observe any immunomodulatory effect with our formulation, we further proceeded with a combination strategy for IBD treatment.

### 3.3 Hybrid lipid hyaluronate-KPV conjugated nanocapsules loaded with teduglutide for combination therapy in IBD

To provide LNC-Ted with anti-inflammatory properties, we first coated LNC-Ted with KPV-C_12_ following the same principle as previous studies coupling gemcitabine C_12_ to lipid nanocapsules [34], coupling KPV-C_12_ onto the surface of our lipid nanocapsules. Following a 7-day daily administration in a murine acute DSS model, we did not observe any effect on pro-inflammatory cytokines (Supplementary Information, Fig. 8S). Hence, we concluded that a different more effective combination would be needed towards an increased anti-inflammatory effect. This was the rationale to tailor the nanoparticles with hyaluronic acid (HA) conjugated with KPV (HA-KPV). We selected HA with a 1 MDa molecular weight (Mw) since it has been demonstrated that this polysaccharide exerts a dualistic Mw-dependent biological response. Indeed, proinflammatory effects are induced by low molecular weight HA (LMw-HA), and conversely, anti-inflammatory and immune suppressive properties are derived from high molecular weight HA (HMw-HA) [18]. Thus, we hypothesized that a high-Mw HA could be used for our therapeutic purposes. Because of its high biocompatibility, hydrophilicity and multivalence, HA is an excellent clinically translatable candidate for drug delivery, possibly resulting in the introduction of rationally designed linkers on its backbone, allowing a disease-responsive drug release. Moreover, HA has already shown beneficial effects in murine colitis by mitigating edema formation, inducing reconstruction of the mucosal barrier, and promoting an anti-inflammatory response [[35], [36], [37]].

**Fig. 4:**
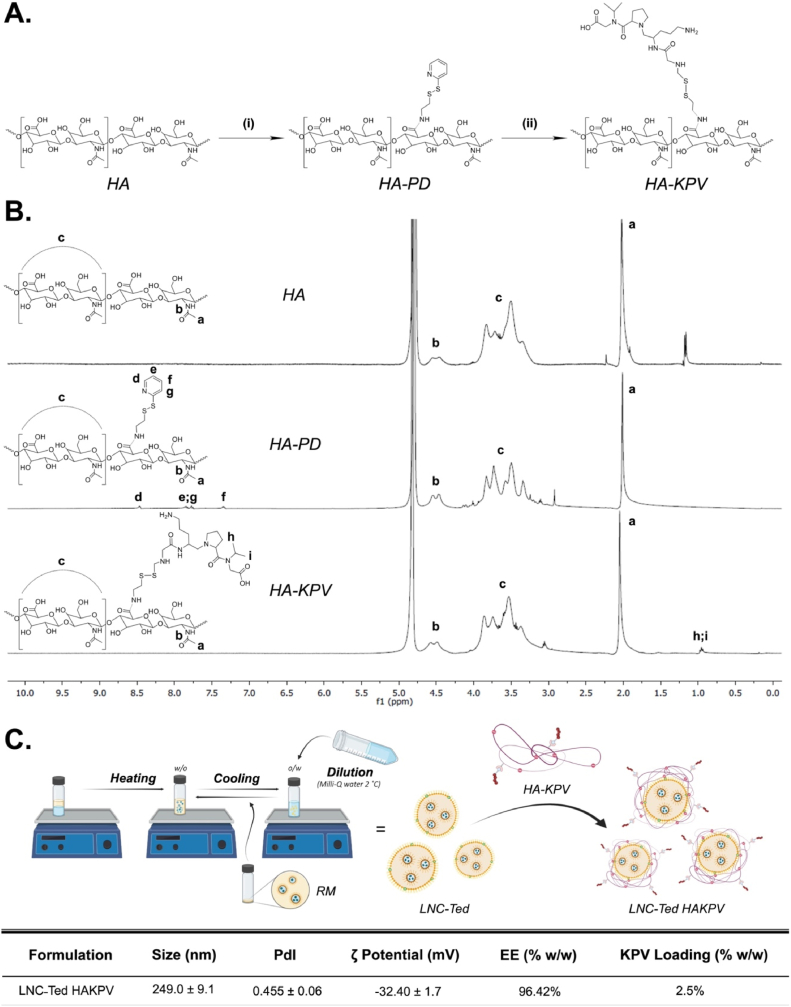
Synthesis and preparation of LNC-Ted HAKPV. (A) Synthetic scheme describing the conjugation strategy used for HA-KPV. (i) DMTMM·Cl, PBS pH = 7.4, 90 min, RT; 2-(pyridyldithio) ethylamine, PBS pH = 7.4, 48 h, RT; (ii) CysKPV, PBS pH = 7.4, 24 h, RT. (B) ^1^H NMR spectra of HA, HA-PD and HA-KPV in D_2_O. (C) Graphical representation of the formulation steps to prepare LNC-Ted HAKPV and the physicochemical characterization of the formulation.

HA-KPV was designed by conjugating KPV to HA with redox-sensitive linking chemistry (Fig. 4A). For the conjugation of KPV, we modified HA with 2-(pyridyldithio) ethylamine using DMTMM·Cl to form HA-PD (2.87% mol/mol functionalization). DMTMM is a morpholine-based reagent that is water-soluble and can be used as an alternative to EDC/NHS chemistry. This type of chemistry, indeed, offers higher aqueous stability of HA over EDC/NHS; it consists of a single step and can be carried out in water at physiological pH values [17]. We conjugated KPV to the HA backbone. For this purpose, we extended the tripeptide sequence with a cysteine, resulting in CysKPV. Next, this peptide was conjugated to HA-PD by redox-sensitive formation. This linking chemistry has been chosen given that oxidative stress plays a key role in the development of intestinal damage in IBD. Indeed, during the active phase of the disease, leukocytes are also involved in an excess induction of oxidative reactions, which remarkably alters the redox equilibrium in the intestinal mucosa, maintaining inflammation by inducing redox-sensitive signaling pathways. ^1^H NMR confirmed the chemical identity of HA-KPV (Fig. 4B). Indeed, the characteristic 2-(pyridyldithio) ethylamine peaks of HA-PD at 8.4, 7.8 and 7.3 ppm disappeared after conjugation with CysKPV, suggesting the displacement of the 2-pyridin thiol in favor of KPV. Moreover, the chemical signature of KPV appeared at 0.9 ppm. The covalent incorporation of the peptide into HA was quantified using a HPLC, resulting in a 2.5% of conjugation, allowing us to reach doses of KPV found to be therapeutically relevant in IBD treatment [16] (Fig. 4D).

Next, we prepared a hybrid lipid hyaluronate-KPV nanoplatform by surface modification of LNC-Ted with HA-KPV. To do so, we slightly modified the lipid composition using 0.12% (w/w) DOTAP instead of 0.26% (w/w) Lipoid to endow LNC-Ted with a positive surface charge (+31.05 ± 1.57 mV, mean ± SD, n = 3). Taking advantage of the anionic charge of HA-KPV, we exploited the coulombic interaction between LNC-Ted and HA-KPV (Fig. 4C). We explored the assembly at different LNC/HA ratios (Supplementary information, Fig. 9S). We selected a ratio of 1:20, allowing for a suitable size and zeta-potential. All the formulations were characterized in terms of size, PdI, and zeta-potential (Supplementary Information, Table 2S), showing consistent and reproducible results for all the analyzed parameters, with an increase in dimension and negative surface charge due to the HA coating. The novel hybrid lipid HA-KPV-conjugated nanoparticles exhibited a nanoparticle size of 250 nm and zeta-potential of −30 mV.

**Fig. 5:**
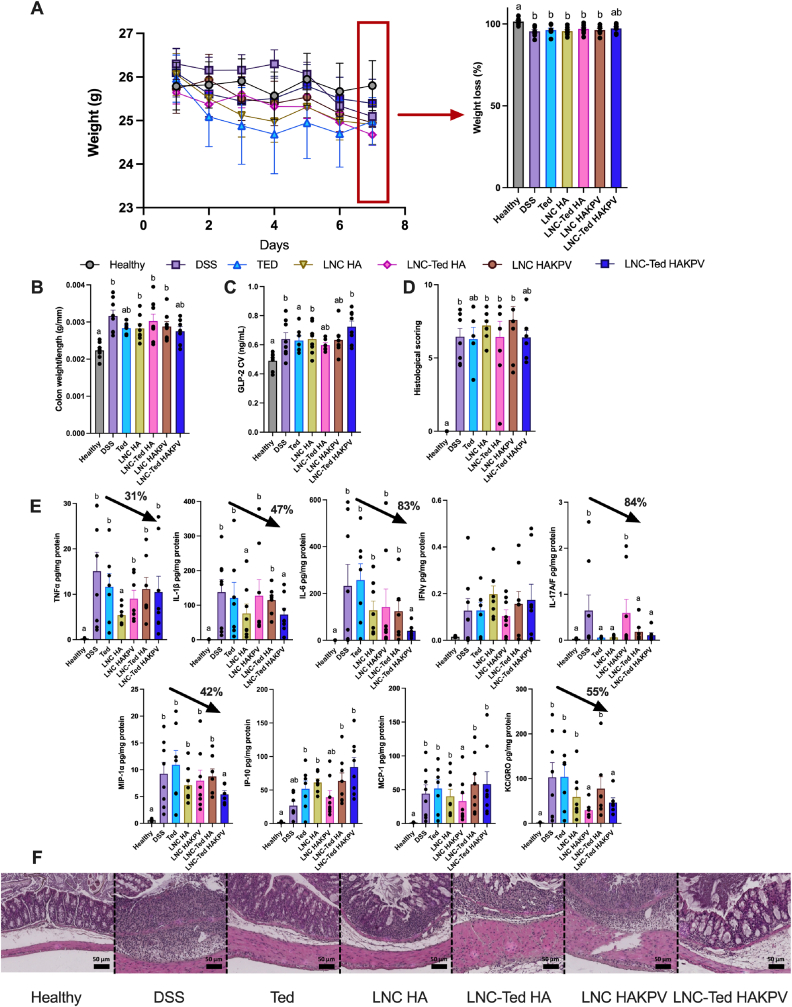
Effect of LNC conjugated with HA or HAKPV (loaded or unloaded with Ted) in an acute DSS-induced murine colitis model. (A) Body weight (g) and body weight loss (%) over the study period (7 days) (n = 7–8). (B) Colon weight/length ratio (n = 7–8). (C) Total GLP-2 plasma levels (ng/mL) (CV: cava vein) (n = 6–8). (D) Histological scoring assessment (n = 7–8). (E) Pro-inflammatory cytokines *TNF-α, IL-1β, IL-6, IFN-γ, IL-*17A/F, and chemokines *KC/GRO, MCP-1, MIP-1α, IP-10* in the colon following acute treatment in a DSS-induced colitis model (n = 6–8). Black arrows represent the reduction in cytokine/chemokine levels (%) with respect to the vehicle-treated DSS group. (F) Representative colon H&E staining images (scale bar: 50 μm). Data with different superscript letters are significantly different (*P < 0.05) according to one-way analysis of variance followed by Tukey's post hoc test (A,B,C) or Kruskal-Wallis test followed by Dunn's post hoc test (D, E) (mean ± SEM).

First, we conducted an acute study to evaluate the anti-inflammatory properties of our combination strategy in an acute DSS model, following a daily administration regimen until the day before sample collection. Notably, in this case, we were not measuring the GLP-2 levels induced by the nanocarriers on the day of sample collection but rather the accumulated levels of GLP-2 upon repeated administration of the different treatments. Our aim was to evaluate our combination therapy in an acute colitis model following consecutive doses of our nanocapsules. We included different treatment groups (e.g., LNC HA, LNC-Ted HA, LNC HAKPV), and we aimed to select from this study the most promising formulation providing both intestinotrophic and anti-inflammatory effects for a subsequent long-term study. Overall, we did not observe significant differences between the LNC-Ted HAKPV group and the vehicle-treated DSS group regarding weight loss (Fig. 5A), colon weight/length ratio (Fig. 5B), GLP-2 levels (Fig. 5C) or histological scoring (Fig. 5D). Additionally, there were no significant differences encountered with the healthy control group (except for GLP-2 levels). This was also the case for the Ted-treated groups in terms of the colon weight/length ratio (Fig. 5B) and histological scoring (Fig. 5D). We evaluated the effect of the formulations on the expression of cytokines/chemokines that were found to be overexpressed in the DSS model according to our previous studies (Supplementary Information, Fig. 3S). Regarding the differences in cytokine/chemokine expression, among the DSS groups receiving treatment, LNC-HA induced a significant reduction in pro-inflammatory TNF-α, IL-1β and IL-17A/F compared to the vehicle-treated DSS group (*P < 0.05) (Fig. 5E). LNC HAKPV exhibited reduced levels of MCP-1 and KC/GRO compared to the DSS-treated control group (*P < 0.05). LNC-Ted HAKPV showed decreased levels of IL-6, IL-1β, IL-17A/F, MIP-1α and KC/GRO compared to the vehicle-treated DSS group (*P < 0.05). These data suggest that HA is actively involved in the anti-inflammatory process, potentiating the effect already observed by LNCs. Indeed, the steric hindrance of HMw-HA leads to its binding with CD44, switching it into its “inactive binding state” and simultaneously preventing endogenous ligand access to TLR-2/4, reducing the infiltration of immune cells and inducing a mild anti-inflammatory effect overall. This is supported by the observed downregulation of TNFα and MCP-1, MIP or IP-10 [[38], [39], [40], [41]].

We acknowledge the fact that other surface modifications (e.g. LNC-HA) could potentially have an effect following long-term treatment. Our aim was to prove that this combined immunomodulatory effect could be tunable and that we could provide a nanoparticle platform toward this end. To reduce the number of animals used in long-term studies, we selected LNC-Ted HAKPV to conduct our proof-of-concept studies. We selected the LNC-Ted HAKPV formulation as a candidate for the long-term treatment because it exhibited increased GLP-2 levels.

**Fig. 6:**
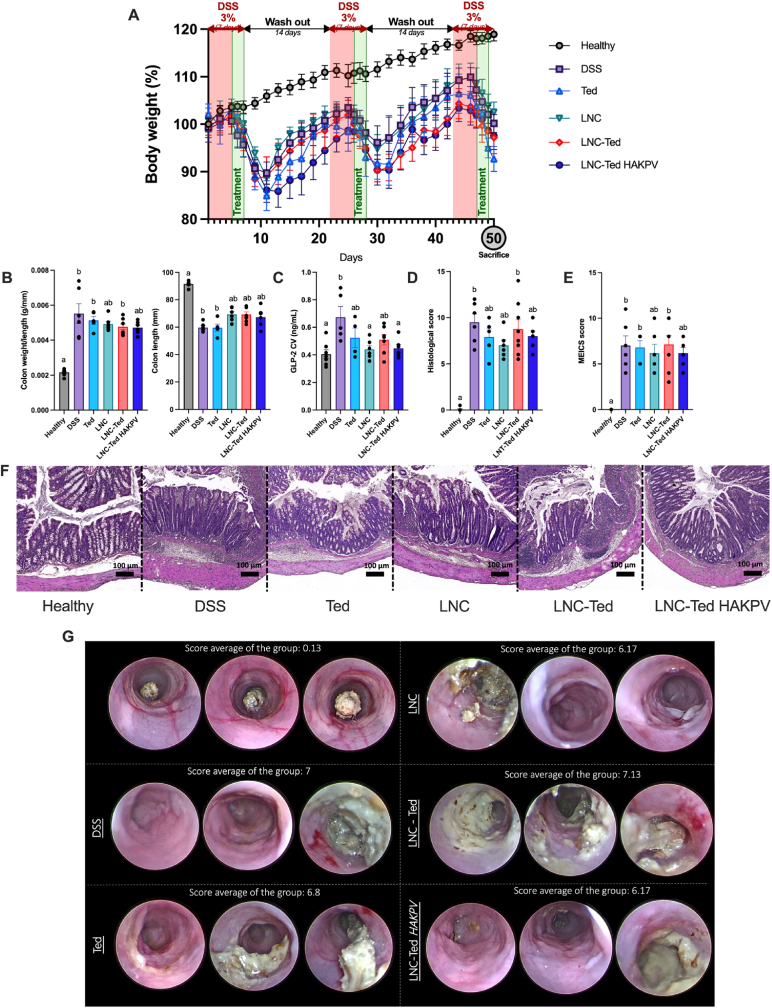
LNC-treated mice exhibited alleviated colitis symptoms in a DSS-induced chronic colitis model. (A) Body weight (%) over the study period (50 days) (n = 5–8). (B) Colon weight/length ratio and colon length (mm) (n = 5–8). (C) Total GLP-2 plasma levels (ng/mL) (cava vein) (n = 5–8 (except n = 4 for Ted)). (D) Histological scoring assessment following a double-blinded analysis per sample (n = 10–16). (E) MEICS scoring assessed by a gastroenterologist (n = 5–8). (F) Representative colon H&E staining images (scale bar: 50 μm). (G) Representative colonoscopy images (n = 3 per condition). Data with different superscript letters are significantly different (*P < 0.05) according to one-way analysis of variance followed by Tukey's post hoc test (C) or Kruskal-Wallis test followed by Dunn's post hoc test (B, D, E) (mean ± SEM).

To reduce a potential tumor-promoting effect, we and others [8] propose a short-term or intermittent use of GLP-2 or its analogs during the course of the disease. In this case, we opted for a 3-day administration regimen at the beginning of the “flare” (worsen state) of each DSS cycle, which starts right after the removal of the DSS solution. We administered a total of 9 doses of Ted, LNC, LNC-Ted or LNC-Ted HAKPV over 50 days, 3 doses per DSS cycle, with 3 DSS cycles in total (Fig. 6). All treated groups exhibited significantly decreased body weight loss compared to the healthy control on Day 50 (*P < 0.05). All LNC-treated groups exhibited an increased colon length and a decreased colon weight/length ratio, although not significantly different when compared to the vehicle-treated DSS group (*P* > 0.05). In the case of this chronic model, we conducted a colonoscopy on the mice at the end of the experiment and evaluated the murine endoscopic index of colitis severity (MEICS) in living mice. A decrease in the histological scoring and MEICS was also observed for these LNC-treated groups, but they were not significantly different compared to the vehicle-treated DSS group (*P* > 0.05).

The increased GLP-2 levels observed upon acute treatment demonstrated that the stimulatory effect of the nanocarriers was preserved despite the hyaluronan-KPV functionalization (Fig. 5C), although these levels were found to be not significant compared to the DSS control group. However, circulating GLP-2 levels were decreased and significantly different from those in the vehicle-treated DSS group (*P < 0.05) after intermittent long-term treatment with LNC and LNC-Ted HAKPV (Fig. 6C). Endogenous GLP-2 plasma concentrations are elevated in IBD patients suggesting an adaptive response to mucosal healing [6]. One might hypothesize that these decreased levels could be the result of improved mucosal healing.

**Fig. 7:**
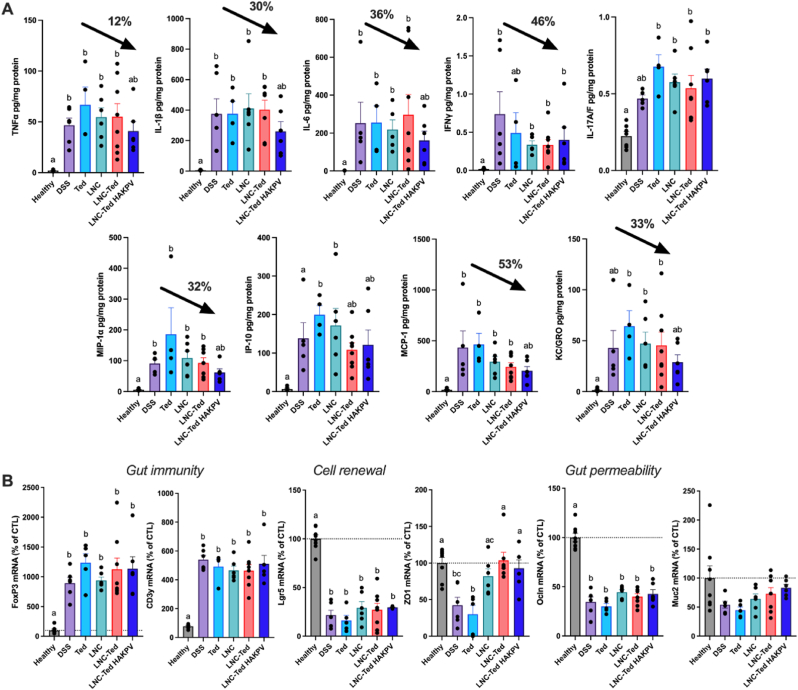
Hybrid lipid hyaluronate-KPV conjugated nanoparticles improved the immunomodulatory response and gut permeability in a DSS-induced chronic colitis model. (A) Pro-inflammatory cytokines *TNF-α, IL-1β, IL-6, IFN-γ, IL-*17A/F and chemokines *MIP-1α, IP-10, MCP-1, KC/GRO* in the colon following chronic treatment in a DSS-induced colitis model (n = 5–8 except for Ted (n = 4)). Black arrows represent the reduction in cytokine/chemokine levels (%) with respect to the DSS control group. (B) mRNA expression of gut immunity (*FoxP*3, *CD3g*), cell renewal (*Lgr5*) and gut permeability (*ZO1*, *Ocln*, *Muc2*) biomarkers in the colon following chronic treatment in a DSS-induced colitis model (n = 5–8; mean ± SEM). Data with different superscript letters are significantly different (*P < 0.05) according to one-way analysis of variance followed by Tukey's post hoc test (*IL-1β, IL-*17A/F*, ZO1, FoxP3, Lgr5, Ocln*) or Kruskal-Wallis test followed by Dunn's post hoc test (*TNF-α, INF-γ, IL-6, KC/GRO, IP-10, MCP-1, MCP-1α, Muc2*) (mean ± SEM).

Consistent with what we found in the acute model, LNC-Ted HAKPV administration decreased the expression of most cytokines/chemokines we measured (Fig. 7A). Notably, the cytokine levels in the chronic model were up to 10 times higher (e.g., MIP-1α and MCP-1) than those in the acute model. Additionally, in the acute model, we administered the formulations for six consecutive days, whereas in the chronic model, we administered them 3 times for 3 consecutive days over 50 days. The mRNA expression of gut immunity (*FoxP*3, *CD3g*), cell renewal (*Lgr5*) and gut permeability (*ZO1*, *Ocln*, *Muc2*) biomarkers in the colon was quantified in the chronic model (Fig. 7B). No differences were observed in the expression of gut immunity and cell renewal markers between the LNC-treated groups and the vehicle-treated DSS group. However, *ZO-1* expression was significantly increased in both the LNC-Ted- and LNC-Ted HAKPV-treated groups (*P < 0.05). We found that *Muc2* mRNA expression was significantly different between LNC-Ted HAKPV (x¯ = 83.31 ± 14.73% of control)-treated mice and those in the DSS control group (x¯ = 52.27 ± 14.54% of control) by the Mann-Whitney test (*P* = 0.0087, Fig. 7B). However, the statistical significance was lost when analyzed by the Kruskal-Wallis test followed by Dunn's post hoc test. No differences were observed with the rest of the treated groups. Despite these encouraging results, we observed no differences by histological or colonoscopy analysis (Fig. 6D–G). Prolonged treatments and/or doses might be required to observe a complete re-epithelization in the chronic pathological setting.

Taken together the decreased GLP-2 levels (Fig. 6C), the decreased cytokine levels (Fig. 7A) and the increase in the expression of gut permeability biomarkers (Fig. 7B), support this nanoparticle platform as a promising tool for combined mucosal healing and immunomodulation in IBD treatment. Compared to previously reported studies, we are providing with an oral delivery alternative for the delivery of two different peptides (teduglutide and KPV) for a combination therapy in IBD treatment. The use of a redox responsive linking chemistry allowed us to target the oxidative stress observed in IBD towards a selective delivery to inflamed regions. HA-PKV conjugates provided with improved anti-inflammatory properties to the nanocarrier (e.g., decreased pro-inflammatory cytokine levels), whereas the lipid nanocapsules encapsulating teduglutide provided with a mucosal healing effect (e.g., increased *ZO-1* and *Muc2* mRNA levels). Altogether, this represents a novel combination therapy in IBD treatment via oral route.

**Fig. 8:**
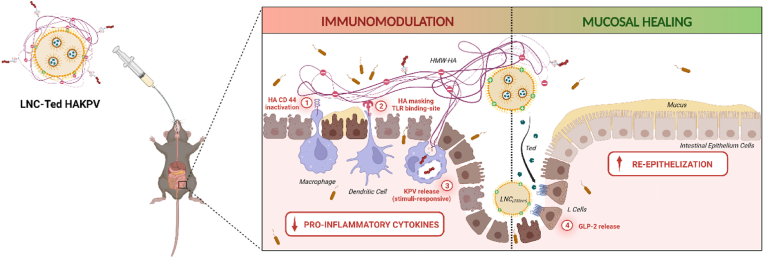
Graphical schematic representation of the proposed LNC-Ted HAKPV mechanism of action in IBD treatment. The efficacy of LNC-Ted HAKPV nanoparticles is in part due to its combined mechanism of (*a*) immunomodulation of HA-KPV based on (1) the inactivation and (2) masking of immune cells from endogenous pro-inflammatory ligands associated with (3) the intracellular release of KPV and (*b*) the mucosal healing effect exerted by LNC-Ted that induce a local release of GLP-2 after interaction with L cells. Created with BioRender.com.

A schematic representation of the proposed mechanism of action of LNC-Ted HAKPV in IBD treatment is depicted in Fig. 8. The high Mw HA conjugated to KPV exerted anti-inflammatory and immune suppressive properties by binding to the CD44 receptor expressed in pro-inflammatory or activated (M1) macrophages (1) and inhibiting their activation by lipopolysaccharides (LPS) by binding to the toll-like receptor-4 (TLR-4) [42] (2). KPV was conjugated to HA via redox-sensitive linking chemistry, allowing the release of KPV, and maximizing the concentrations of the peptide, at the inflammatory sites (3). Altogether, HA-KPV conjugates would provide with anti-inflammatory properties at the inflamed intestinal sites. On the other hand, lipid nanocapsules encapsulating GLP-2 would provide with increased GLP-2 levels providing with mucosal healing properties and inducing intestinal re-epithelization.

## 4 Conclusions

Current available IBD treatments act by inhibiting inflammation. Rebuilding the mucosa without immunosuppression has been described as a future goal of IBD treatment. Herein, we provide with a nanoparticle platform in which we could tune the immunomodulatory effect of the formulation based on the stage and/or needs of the disease. The pharmacological effect exerted by our nanocarriers induced a sufficient stimulus by providing mucosal healing upon intermittent administration of our formulation for 3 consecutive days, 3 times over 50 days, with 9 doses in total. The nanocarriers induced (or did not induce) immunosuppression depending on the presence (or absence) of a hyaluronan-KPV functionalization. This strategy holds promise as a nanoparticle platform for combined mucosal healing and immunomodulation in IBD treatment.

## Data availability

All data relevant to the study are included in the article or uploaded as supplementary information. Complementary data that support the findings of this study are available from the corresponding author, A.B., upon reasonable request.

## CRediT authorship contribution statement

**Valentina Marotti:** Conceptualization, Methodology, Formal analysis, Investigation, Writing – original draft, Writing – review & editing, Visualization. **Yining Xu:** Conceptualization, Methodology, Formal analysis, Investigation, Writing – original draft. **Cécilia Bohns Michalowski:** Conceptualization, Methodology, Formal analysis, Investigation. **Wunan Zhang:** Investigation. **Inês Domingues:** Investigation. **Hafsa Ameraoui:** Investigation. **Tom G. Moreels:** Methodology, Investigation, Writing – review & editing. **Pieter Baatsen:** Methodology, Investigation, Writing – review & editing. **Matthias Van Hul:** Conceptualization, Methodology. **Giulio G. Muccioli:** Conceptualization, Writing – review & editing. **Patrice D. Cani:** Conceptualization, Writing – review & editing. **Mireille Alhouayek:** Conceptualization, Writing – review & editing. **Alessio Malfanti:** Conceptualization, Methodology, Formal analysis, Investigation, Writing – original draft, Writing – review & editing, Supervision. **Ana Beloqui:** Conceptualization, Methodology, Formal analysis, Investigation, Writing – original draft, Writing – review & editing, Visualization, Supervision, Project administration, Funding acquisition.

## Declaration of competing interest

A.B., Y.X. and P.D.C. are inventors of a patent application (WO/2020/254083A1 - Lipid nanocapsules charged with incretin mimetics). P.D.C. is coinventor on patent applications dealing with the use of specific bacteria and components in the treatment of different diseases. P.D.C. was co-founder of The Akkermansia Company SA and Enterosys.
