# Supplementary material for: A nanoparticle platform for combined mucosal healing and immunomodulation in inflammatory bowel disease treatment
Source: Bioact Mater. Author manuscript; Available in PMC 2024 Feb 1. (PMC10582360; doi:10.1016/j.bioactmat.2023.09.014)
Supplement: Supplementary material [file EMS189554-supplement-Supplementary_material.pdf]

A nanoparticle platform towards combined mucosal healing and immunomodulation in inflammatory bowel disease treatment

Valentina Marottia[a1], Yining Xu[a1], Cécilia Bohns Michalowski[a1], Wunan Zhang[a], Inês Domingues[a], Hafsa Ameraoui[b], Tom G. Moreels[c,d], Pieter Baatsen[e], Matthias Van Hul[f], Giulio G. Muccioli[b], Patrice D. Cani[f,g,h], Mireille Alhouayek[b], Alessio Malfanti[a], Ana Beloqui[a,h]*

[a]UCLouvain, Université catholique de Louvain, Louvain Drug Research Institute, Advanced Drug Delivery and Biomaterials, 1200 Brussels, Belgium

[b]UCLouvain, Université catholique de Louvain, Louvain Drug Research Institute, Bioanalysis and Pharmacology of Bioactive Lipids, 1200 Brussels, Belgium

[c]UCLouvain, Université catholique de Louvain, Institute of Experimental and Clinical Research, Laboratory of Hepato-Gastroenterology, 1200 Brussels, Belgium

[d]Cliniques universitaires Saint-Luc, Department of Hepato-Gastroenterology, Brussels, Belgium

[e]EM-platform, VIB Bio Imaging Core, KU Leuven, Campus Gasthuisberg, Herestraat 49, 3000 Leuven

[f]UCLouvain, Université catholique de Louvain, Louvain Drug Research Institute, Metabolism and Nutrition Group, 1200 Brussels, Belgium

[g]UCLouvain, Institute of Experimental and Clinical Research, 1200 Brussels, Belgium

[h]WEL Research Institute, avenue Pasteur, 6, 1300 Wavre, Belgium

[1]Equal contribution
*Correspondence to:
Dr. Ana Beloqui
Université catholique de Louvain, Louvain Drug Research Institute, Advanced Drug Delivery and Biomaterials, 1200 Brussels, Belgium
Tel +32 (0)27647320. E-mail: ana.beloqui@uclouvain.be

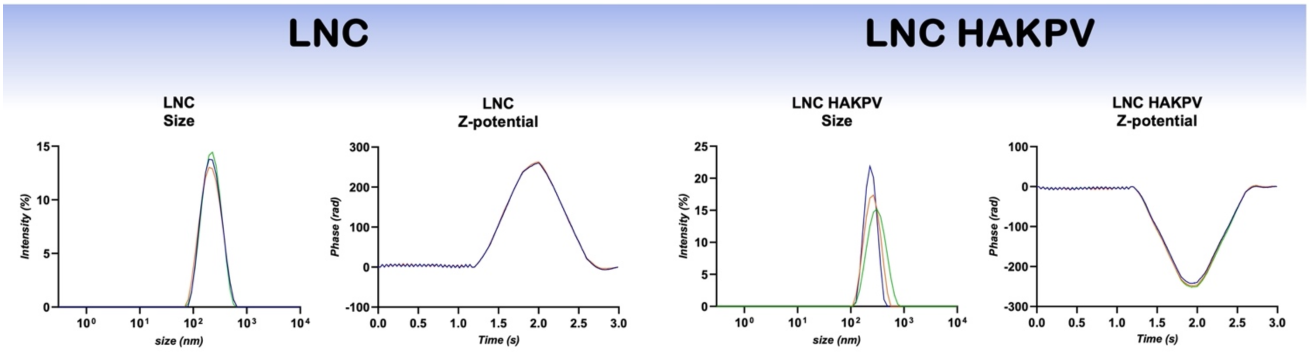

LNC

Size (nm)	PdI	Z-Potential (mV)
203.9 ± 5.3	0.1593 ± 0.04	+38.2 ± 0.42

LNC HAKPV

Size (nm)	PdI	Z-Potential (mV)
261.4 ± 6.2	0.3227 ± 0.07	-32.7 ± 1.1

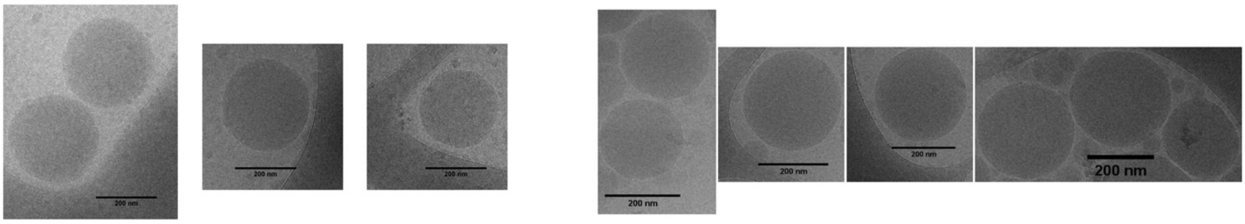

Figure 1S. Representative DLS graphs of the size and zeta potential of LNC and LNC HAKPV nanocapsules and corresponding cryo-TEM representative images (mean ± SD, n = 3).

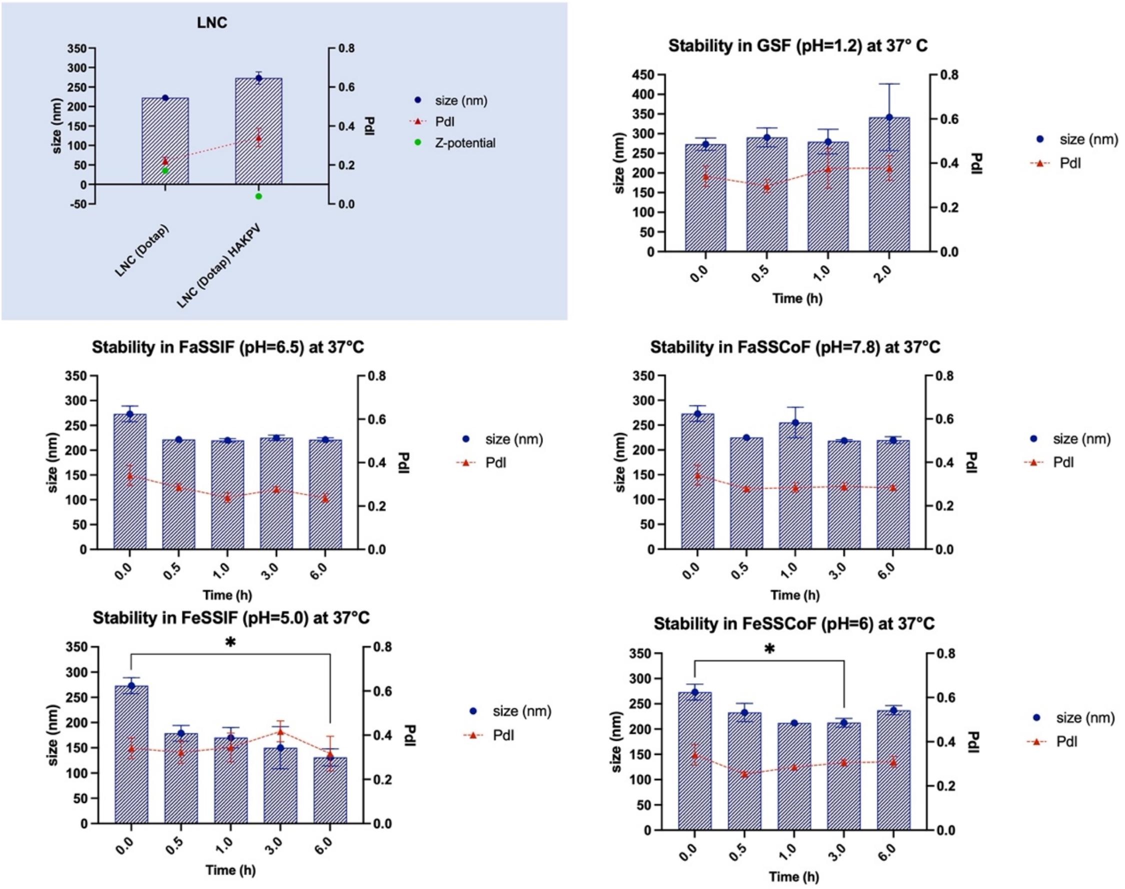

Figure 2S. **In vitro stability studies in biomimetic media.** Significant differences (*$P<0.05$) were calculated according to Kruskal-Wallis test followed by Dunn's post hoc test ($n = 3$; mean ± SEM).

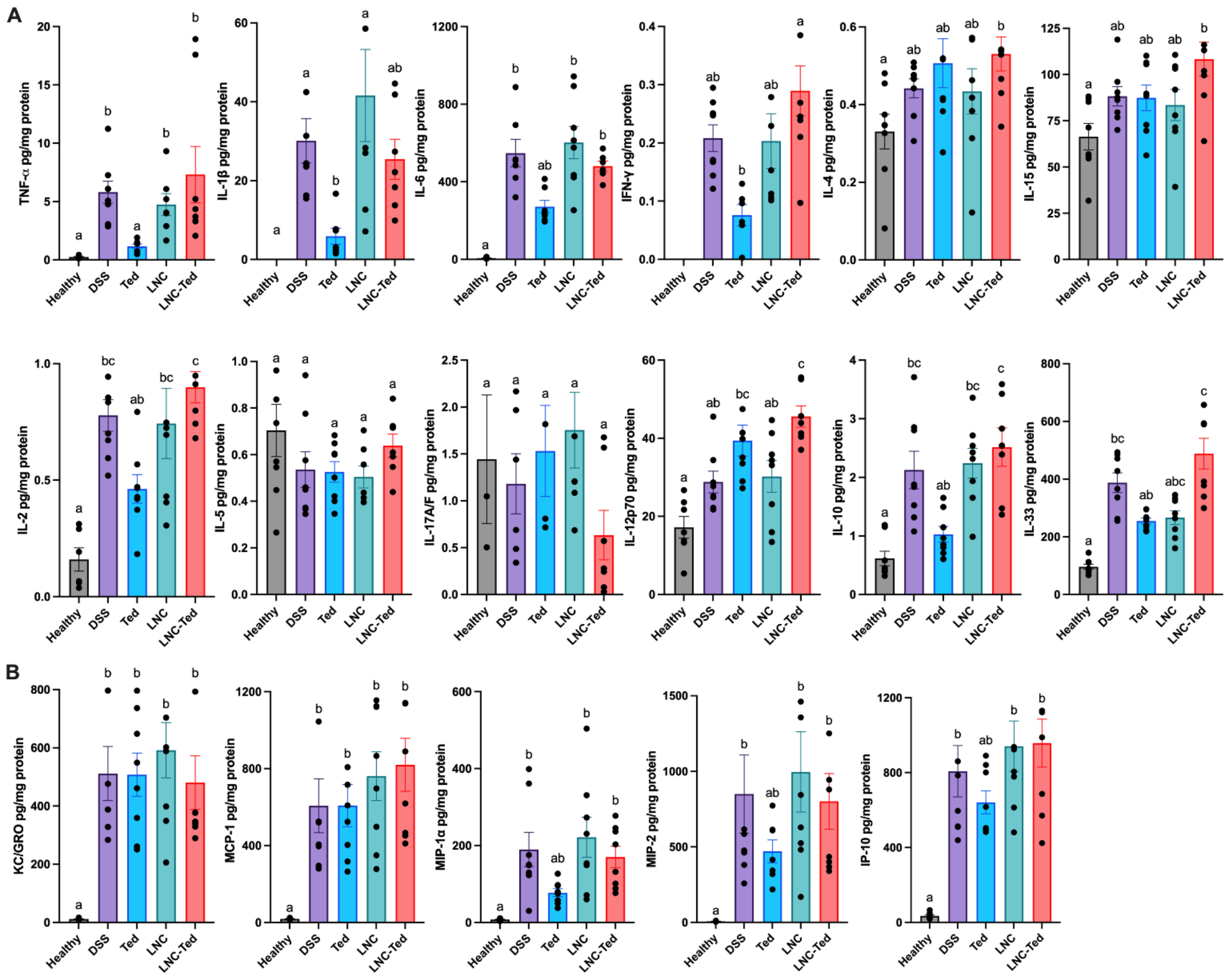

Figure 3S. **A) Pro-inflammatory cytokines TNF-α, IL-1β, IL-6, IFN-γ, IL-4, IL-15, IL-2, IL-5, IL-17A/F, IL-12p70, IL-10 and IL-33 and B) chemokines KCR/GRO, MCP-1, MIP-1α, MIP-2, IP-10 in the colon following an acute treatment in a DSS-induced colitis model.** Data with different superscript letters are significantly different (*P<0.05). Significant differences (*P<0.05) were calculated according to one-way analysis of variance followed by Tukey's post hoc test (*IL-15, IFN-γ, IL-12p70, IL-2, IL-4, IL-5*) or Kruskal-Wallis test followed by Dunn's post hoc test (*IL-17A/F, IL-33, IP-10, MCP-1, MIP-1α, MIP-2, IL-10, IL-1β, IL-6, KC/GRO, TNF-*α) (n = 6-8; mean ± SEM, except for IFN-γ and IL-1β where the levels in the healthy group were undetectable and Il-17A-F were levels in Healthy (n = 3) and Ted (n = 4) groups were detected only in some mice). Cytokine levels for IL-27 and IL-9 were too low and thus, have not been included within this graph.

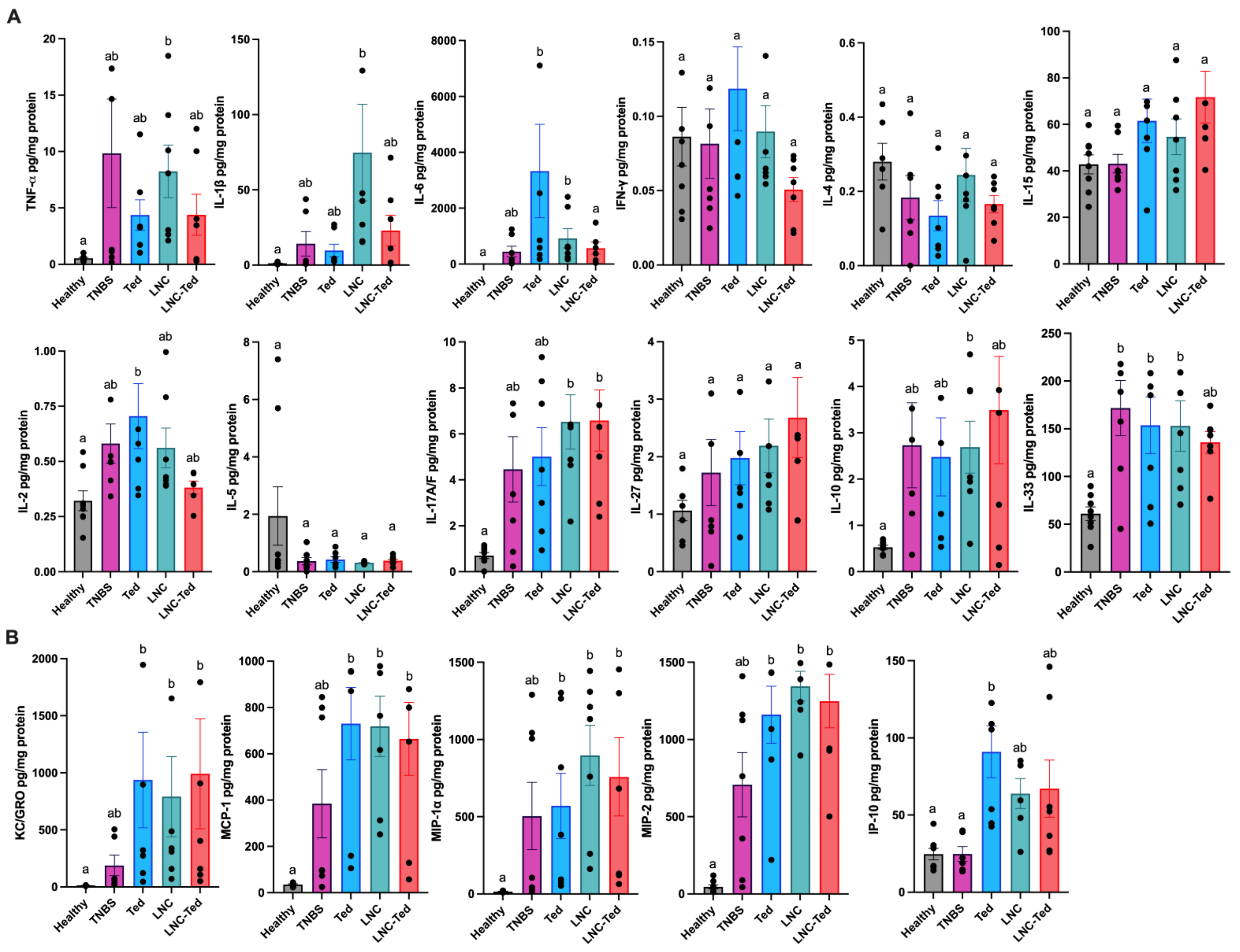

Figure 4S. A) Pro-inflammatory cytokines TNF-α, IL-1β, IL-6, IFN-γ, IL-4, IL-15, IL-2, IL-5, IL-17A/F, IL-12p70, IL-10 and IL-33 and B) chemokines KC/GRO, MCP-1, MIP-1α, MIP-2, IP-10 in the colon following an acute treatment in a TNBS-induced colitis model. Data with different superscript letters are significantly different (*P<0.05). Significant differences (*P<0.05) were calculated according to one-way analysis of variance followed by Tukey's post hoc test (*IL-15, IL-27, IL-33, IL-4*) or Kruskal-Wallis test followed by Dunn's post hoc test (*IFN-γ, IL-2, IL-5, IL-17A/F, IL-33, IP-10, MCP-1, MIP-1α, MIP-2, IL-10, IL-1β, IL-6, KC/GRO, TNF*-α) (n = 6-8; mean ± SEM). Cytokine levels for IL-9 were undetectable and IL-12p70 levels were detected only in some mice and thus, have not been included within this graph.

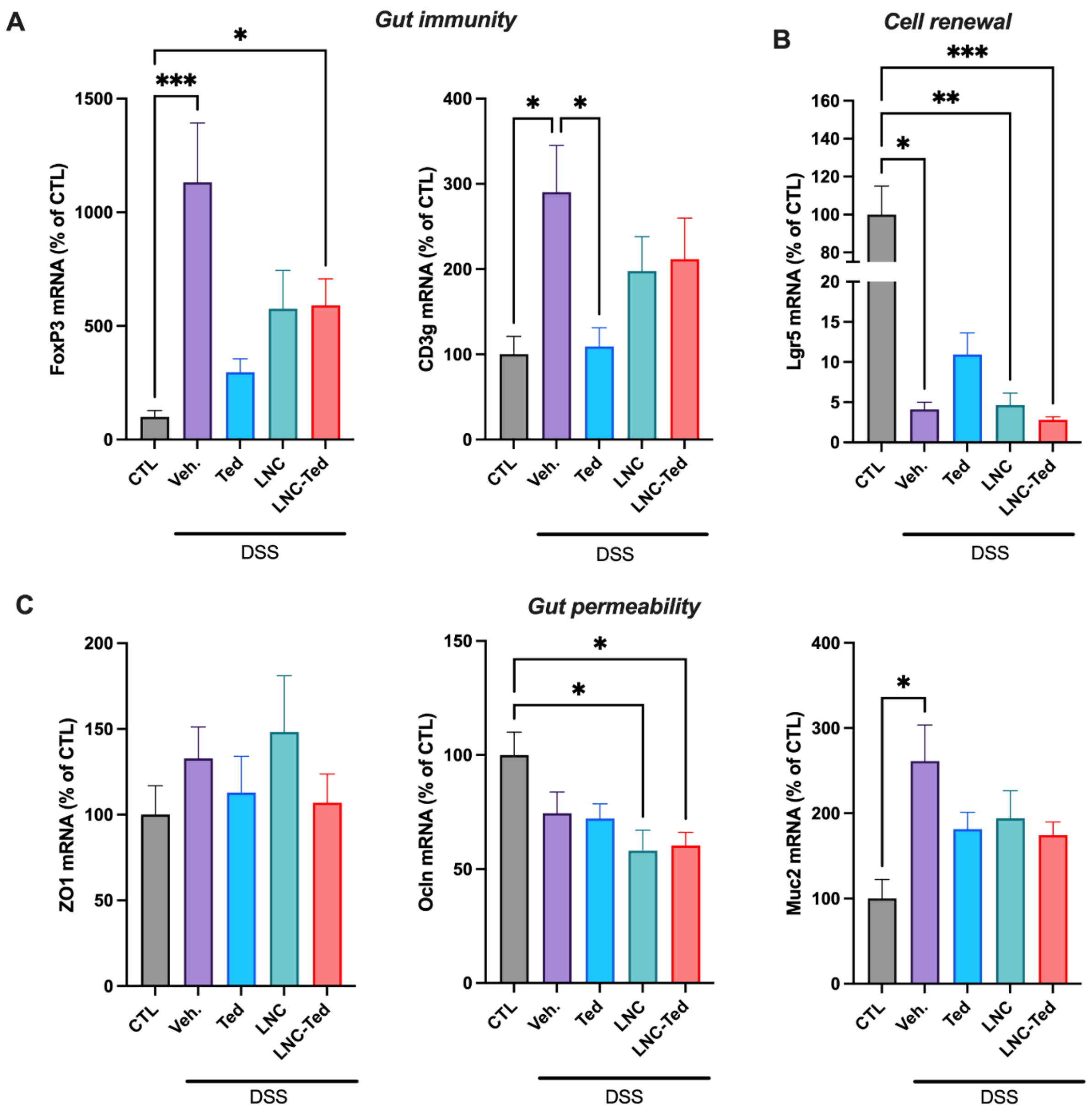

Figure 5S. **mRNA expression of gut immunity (*FoxP*3, *CD3g*) (A), cell renewal (*Lgr5*) (B) and gut permeability (*ZO1*, *Ocln*, *Muc2*) (C) biomarkers in the colon following an acute treatment in a DSS-induced colitis model.** Significant differences (*P<0.05) were calculated according to one-way analysis of variance followed by Tukey's post hoc test (*CD3g*, *ZO1*, *Ocln*) or Kruskal-Wallis test followed by Dunn's post hoc test (*FoxP3*, *Muc2*, *Lgr5*) (n = 6-8; mean ± SEM).

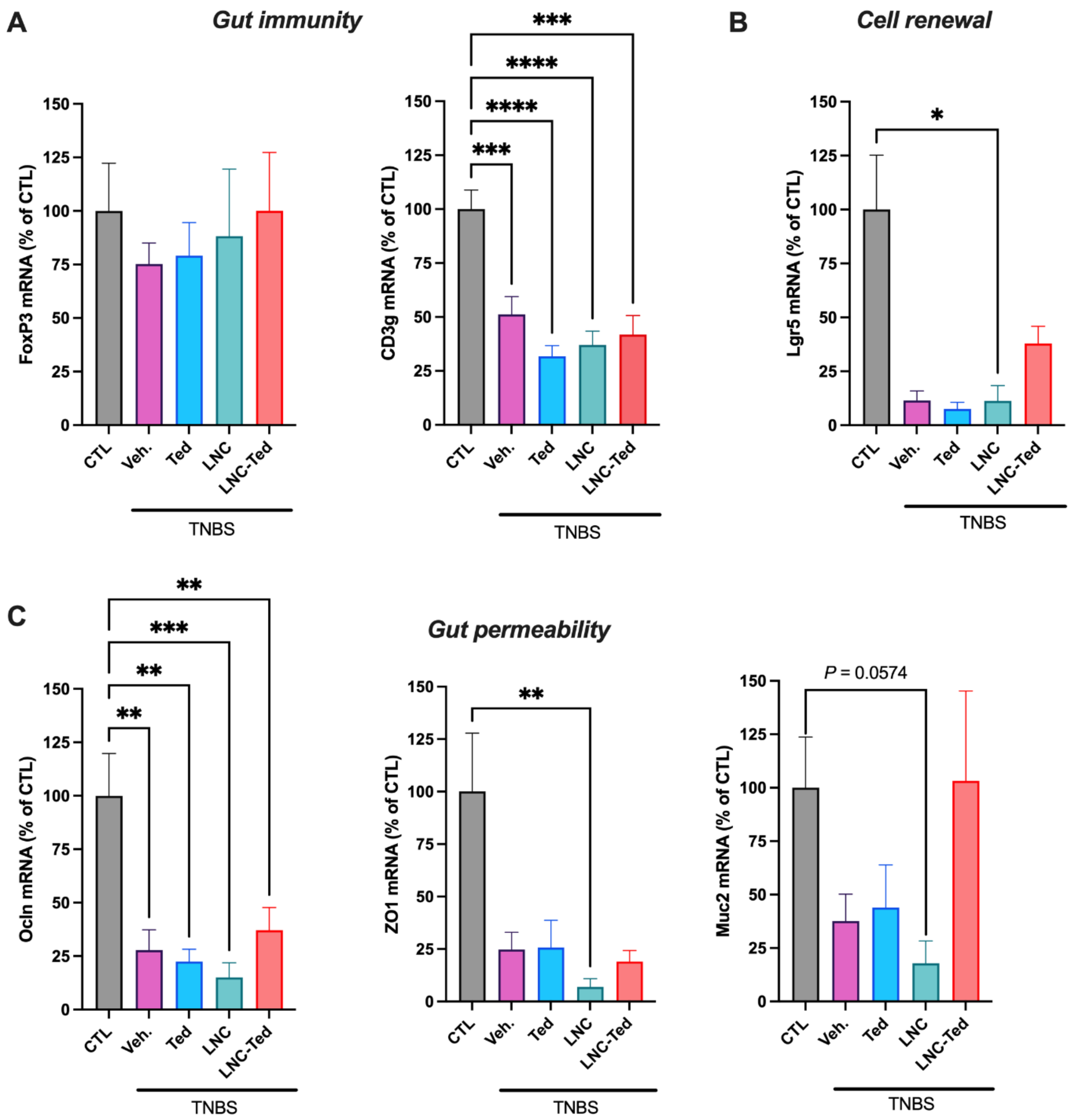

Figure 6S. **mRNA expression of gut immunity (*FoxP*3, *CD3g*) (A), cell renewal (*Lgr5*) (B) and gut permeability (*ZO1*, *Ocln*, *Muc2*) (C) markers in the colon following an acute treatment in a TNBS-induced colitis model.** Significant differences (*P<0.05) were calculated according to one-way analysis of variance followed by Tukey's post hoc test (*FoxP3*, *CD3g*, *Ocln*) or Kruskal-Wallis test followed by Dunn's post hoc test (*Muc2*, *ZO1*, *Lgr5*) (n = 6-7; mean ± SEM).

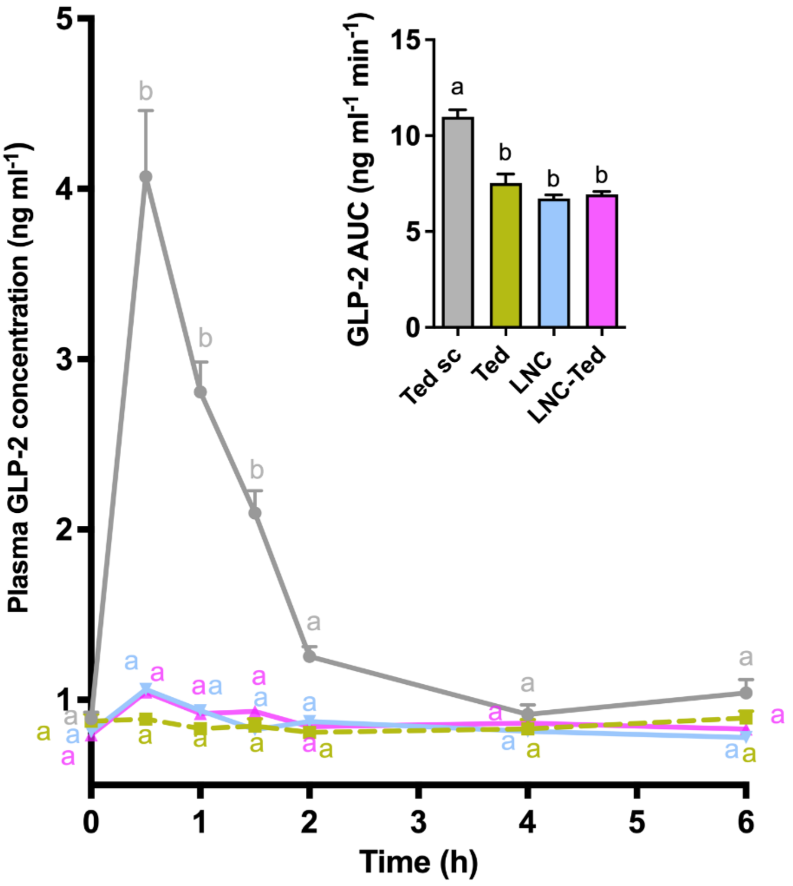

Figure 7S. Teduglutide and total GLP-2 plasma concentration (ng/mL) and area under the curve (AUC) ng/mg·mL in healthy mice (n = 6-8; mean ± SEM). Data with different superscript letters are significantly different (*$P<0.05$) according to two-way analysis (plasma GLP-2 concentrations) or one-way analysis of variance (AUC) followed by Tukey's post hoc.

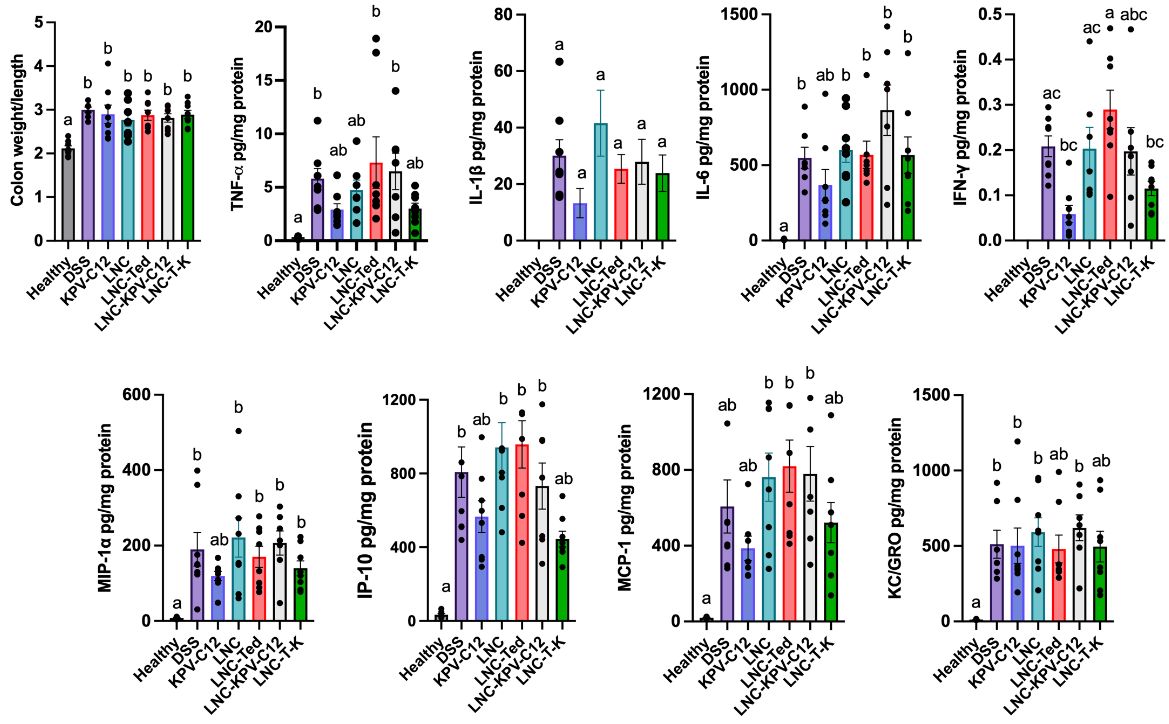

Figure 8S. Effect of LNC coated with KPV-C_{12} (30 µL of a 20 mg/mL KPV-C_{12} solution) (loaded or unloaded with Ted) in an acute DSS-induced murine colitis model, following a daily 7-day administration (n = 7-8; mean ± SEM) (LNC-T-K: LNC-Ted coated with KPV-C_{12}). Control groups Healthy, DSS, LNC and LNC-Ted are the same as the ones in Figure 2 and Figure 3S as these studies were conducted together. Significant differences (*$P<0.05$) were calculated according to one-way analysis of variance followed by Tukey's post hoc test or Kruskal-Wallis test followed by Dunn's post hoc test.

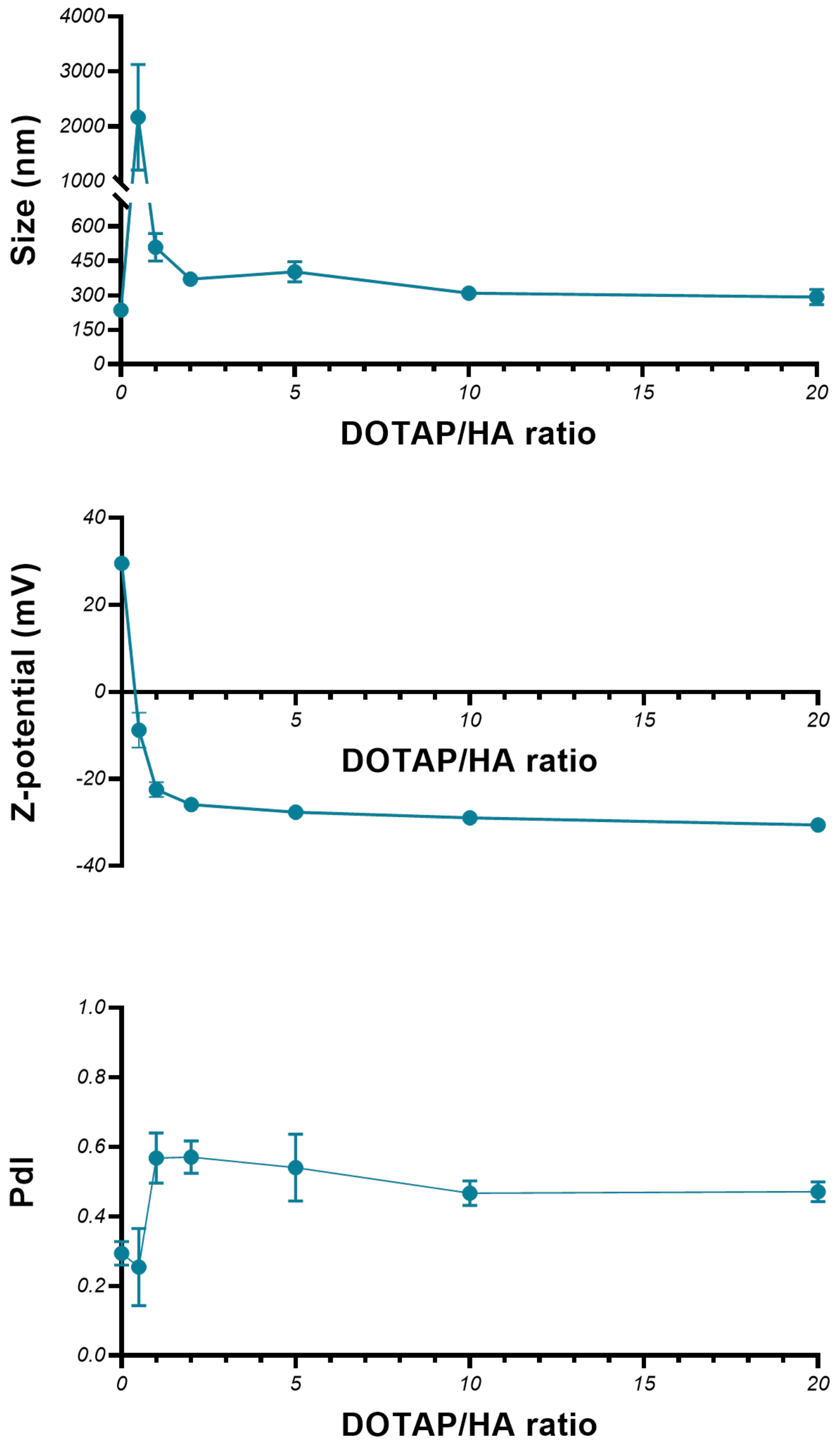

Figure 9S. Physico-chemical characterization of the different tested ratio DOTAP:HA (ratio 1: 0.5-20). The ratio 1: 20 was selected since it showed the lowest values in terms of size, PdI and Z-potential (mean ± SD, n=3).

Table 1S. Final composition of LNCs differing in surface charge. Negatively-charged LNCs were obtained using 13.4 mg Lipoid. Positively-charged LNCs were prepared using 6.0 mg DOTAP. For blank nanoparticles, 150 µL PBS solution was used instead of 150 µL Teduglutide solution (10 mg/mL; pH=10).

		Surface Negatively-charged LNC	Surface Positively-charged LNC
LNC (200 nm)	Lipoid	13.4 mg	/
	DOTAP	/	6.0 mg
	Labrafac	769.5 mg	769.5 mg
	Peceol	85.5 mg	85.5 mg
	Kolliphor	120.0 mg	120.0 mg
	NaCl	50.0 mg	50.0 mg
	Milli-Q water	1.025 mL	1.025 mL
RM	Labrafac	250 mg	250 mg
	Span 85	150 mg	150 mg
	Teduglutide *(In PBS 10mg/mL pH=10.0)*	150 µL	150 µL

Table 2S. Size, PdI and Z-potential of the different tested formulations (mean ± SD, n=3-6).

Formulation	Size (nm)	PdI	Z-Potential (mV)
LNC	218.8 ± 8.90	0.159 ± 0.03	-16.68 ± 0.76
LNC-HA	263.9 ± 23.2	0.418 ± 0.07	-30.90 ± 0.89
LNC-Ted	217.5 ± 15.9	0.189 ± 0.04	-17.49 ± 1.88
LNC-Ted HA	287.6 ± 36.4	0.433 ± 0.13	-31.40 ± 1.19
LNC-Ted HAKPV	249.0 ± 9.09	0.455 ± 0.06	-30.70 ± 1.69
LNC + Ted	218.4 ± 11.8	0.163 ± 0.03	-16.20 ± 1.73

Table 3S. Composition of biomimetic intestinal fluids.

Composition	GSF (mM)	FaSSIF-V2 (mM)	FeSSIF-V2 (mM)	FaSSCoF (mM)	FeSSCoF (mM)
Sodium taurocholate	/	3	10	0.15	0.6
Lecithin	/	0.2	2	0.3	0.5
Glycerol monooleate	/	/	5	/	/
Sodium oleate	/	/	0.8	0.1	0.2
Sodium	/	106	218	/	/
Chloride		69	125	/	/
Tris	/	/	/	45	31
Sodium hydroxide	/	/	/	120	34
Maleic acid	/	19	55	76	30
Pepsin	/	/	/	/	/
HCl	q.s.f.				
pH	1.2	6.5	5.8	7.8	6.0
