# Supplementary material for: A nanoparticle platform for combined mucosal healing and immunomodulation in inflammatory bowel disease treatment
Source: Bioact Mater. 2023 Oct 11;32:206–21. doi: 10.1016/j.bioactmat.2023.09.014 (PMC10582360; doi:10.1016/j.bioactmat.2023.09.014)
Supplement: Supplementary file 1 [file mmc1.docx]

**A nanoparticle platform towards combined mucosal healing and immunomodulation in inflammatory bowel disease treatment**

Valentina Marottia^a1^, Yining Xu^a1^, Cécilia Bohns Michalowski^a1^, Wunan Zhang^a^, Inês Domingues^a^, Hafsa Ameraoui^b^, Tom G. Moreels^c,d^, Pieter Baatsen^e^, Matthias Van Hul^f^, Giulio G. Muccioli^b^, Patrice D. Cani^f,g,h^, Mireille Alhouayek^b^, Alessio Malfanti^a^, Ana Beloqui^a,h^*

^a^UCLouvain, Université catholique de Louvain, Louvain Drug Research Institute, Advanced Drug Delivery and Biomaterials, 1200 Brussels, Belgium

^b^UCLouvain, Université catholique de Louvain, Louvain Drug Research Institute, Bioanalysis and Pharmacology of Bioactive Lipids, 1200 Brussels, Belgium

^c^UCLouvain, Université catholique de Louvain, Institute of Experimental and Clinical Research*,* Laboratory of Hepato-Gastroenterology, 1200 Brussels, Belgium

^d^Cliniques universitaires Saint-Luc, Department of Hepato-Gastroenterology, Brussels, Belgium

^e^EM-platform, VIB Bio Imaging Core, KU Leuven, Campus Gasthuisberg, Herestraat 49, 3000 Leuven

^f^UCLouvain, Université catholique de Louvain, Louvain Drug Research Institute, Metabolism and Nutrition Group, 1200 Brussels, Belgium

^g^UCLouvain, Institute of Experimental and Clinical Research, 1200 Brussels, Belgium

^h^WEL Research Institute, avenue Pasteur, 6, 1300 Wavre, Belgium

	

^1^Equal contribution

*Correspondence to:

Dr. Ana Beloqui 

Université catholique de Louvain, Louvain Drug Research Institute, Advanced Drug Delivery and Biomaterials, 1200 Brussels, Belgium

Tel +32 (0)27647320. E-mail: ana.beloqui@uclouvain.be

**Figure 1S**. Representative DLS graphs of the size and zeta potential of LNC and LNC HAKPV nanocapsules and corresponding cryo-TEM representative images (mean ± SD, n = 3).

**Figure 2S**. **In vitro stability studies in biomimetic media.** Significant differences (*P<0.05) were calculated according to Kruskal-Wallis test followed by Dunn’s post hoc test (n = 3; mean ± SEM).

**Figure 3S**. **A)** **Pro-inflammatory cytokines TNF-**α**, IL-1**β**, IL-6, IFN-**γ**,  IL-4, IL-15, IL-2, IL-5, IL-17A/F, IL-12p70, IL-10 and IL-33 and B) chemokines KCR/GRO, MCP-1, MIP-1**α**, MIP-2, IP-10 in the colon following an acute treatment in a DSS-induced colitis model.** Data with different superscript letters are significantly different (*P<0.05). Significant differences (*P<0.05) were calculated according to one-way analysis of variance followed by Tukey’s post hoc test (*IL-15, IFN-*γ*, IL-12p70, IL-2, IL-4, IL-5*) or Kruskal-Wallis test followed by Dunn’s post hoc test (*IL-17A/F, IL-33, IP-10, MCP-1, MIP-1*α*, MIP-2, IL-10, IL-1*β*, IL-6, KC/GRO, TNF-*α) (n = 6-8; mean ± SEM, except for IFN-γ and IL-1β where the levels in the healthy group were undetectable and Il-17A-F were levels in Healthy (n = 3) and Ted (n = 4) groups were detected only in some mice). Cytokine levels for IL-27 and IL-9 were too low and thus, have not been included within this graph.

**Figure 4S**. **A)** **Pro-inflammatory cytokines TNF-**α**, IL-1**β**, IL-6, IFN-**γ**,  IL-4, IL-15, IL-2, IL-5, IL-17A/F, IL-12p70, IL-10 and IL-33 and B) chemokines KC/GRO, MCP-1, MIP-1**α**, MIP-2, IP-10 in the colon following an acute treatment in a TNBS-induced colitis model.** Data with different superscript letters are significantly different (*P<0.05). Significant differences (*P<0.05) were calculated according to one-way analysis of variance followed by Tukey’s post hoc test (*IL-15, IL-27, IL-33, IL-4*) or Kruskal-Wallis test followed by Dunn’s post hoc test (*IFN-*γ*, IL-2, IL-5,* *IL-17A/F, IL-33, IP-10, MCP-1, MIP-1*α*, MIP-2, IL-10, IL-1*β*, IL-6, KC/GRO, TNF-*α) (n = 6-8; mean ± SEM). Cytokine levels for IL-9 were undetectable and IL-12p70 levels were detected only in some mice and thus, have not been included within this graph.

**Figure 5S**. **mRNA expression of gut immunity (*****FoxP*****3,** ***CD3g*****) (A), cell renewal (*****Lgr5*****) (B) and gut permeability (*****ZO1*****,** ***Ocln*****,** ***Muc2*****) (C) biomarkers in the colon following an acute treatment in a DSS-induced colitis model.** Significant differences (*P<0.05) were calculated according to one-way analysis of variance followed by Tukey’s post hoc test (*CD3g*, *ZO1*, *Ocln*) or Kruskal-Wallis test followed by Dunn’s post hoc test (*FoxP3*, *Muc2*, *Lgr5*) (n = 6-8; mean ± SEM).

**Figure 6S**. **mRNA expression of gut immunity (*****FoxP*****3,** ***CD3g*****) (A), cell renewal (*****Lgr5*****) (B) and gut permeability (*****ZO1*****,** ***Ocln*****,** ***Muc2*****) (C) markers in the colon following an acute treatment in a TNBS-induced colitis model.** Significant differences (*P<0.05) were calculated according to one-way analysis of variance followed by Tukey’s post hoc test (*FoxP3*, *CD3g*, *Ocln*) or Kruskal-Wallis test followed by Dunn’s post hoc test (*Muc2*, *ZO1*, *Lgr5*) (n = 6-7; mean ± SEM).

**Figure 7S**. Teduglutide and total GLP-2 plasma concentration (ng/mL) and area under the curve (AUC) ng/mg⋅mL in healthy mice (n = 6-8; mean ± SEM). Data with different superscript letters are significantly different (**P*<0.05) according to two-way analysis (plasma GLP-2 concentrations) or one-way analysis of variance (AUC) followed by Tukey’s post hoc. 

**Figure 8S**. Effect of LNC coated with KPV-C_12_ (30 µL of a 20 mg/mL KPV-C_12_ solution) (loaded or unloaded with Ted) in an acute DSS-induced murine colitis model, following a daily 7-day administration (n = 7-8; mean ± SEM) (LNC-T-K: LNC-Ted coated with KPV-C_12_). Control groups Healthy, DSS, LNC and LNC-Ted are the same as the ones in Figure 2 and Figure 3S as these studies were conducted together. Significant differences (*P<0.05) were calculated according to one-way analysis of variance followed by Tukey’s post hoc test or Kruskal-Wallis test followed by Dunn’s post hoc test.

**Figure 9S.** Physico-chemical characterization of the different tested ratio DOTAP:HA (ratio 1: 0.5-20). The ratio 1: 20 was selected since it showed the lowest values in terms of size, PdI and Z-potential (mean ± SD, n=3). 

  

**Table 1S.** Final composition of LNCs differing in surface charge. Negatively-charged LNCs were obtained using 13.4 mg Lipoid. Positively-charged LNCs were prepared using 6.0 mg DOTAP. For blank nanoparticles, 150 µL PBS solution was used instead of 150 µL Teduglutide solution (10 mg/mL; pH=10).

**Table 2S.** Size, PdI and Z-potential of the different tested formulations (mean ± SD, n=3-6).

**Table 3S. Composition of biomimetic intestinal fluids.**

| Composition | GSF (mM) | FaSSIF-V2  (mM) | FeSSIF-V2  (mM) | FaSSCoF (mM) | FeSSCoF (mM) |
| --- | --- | --- | --- | --- | --- |
| Sodium taurocholate | / | 3 | 10 | 0.15 | 0.6 |
| Lecithin | / | 0.2 | 2 | 0.3 | 0.5 |
| Glycerol monooleate | / | / | 5 | / | / |
| Sodium oleate | / | / | 0.8 | 0.1 | 0.2 |
| Sodium | / | 106 | 218 | / | / |
| Chloride |  | 69 | 125 | / | / |
| Tris | / | / | / | 45 | 31 |
| Sodium hydroxide | / | / | / | 120 | 34 |
| Maleic acid | / | 19 | 55 | 76 | 30 |
| Pepsin | / | / | / | / | / |
| HCl | q.s.f. |  |  |  |  |
| pH | 1.2 | 6.5 | 5.8 | 7.8 | 6.0 |
